# Structure and sequence characteristics of 5′-stem-loop 1 modulate the escape from nsp1-mediated repression in SARS-CoV-2 variants

**DOI:** 10.1093/nar/gkag364

**Published:** 2026-04-28

**Authors:** Julian B Schoth, Inge Schwedt, Susanne Philipp, Sabrina Toews, Leonie Schübel, Anna Wacker, Harald Schwalbe, Julia E Weigand

**Affiliations:** Department of Pharmacy, Institute of Pharmaceutical Chemistry, Marburg University, Marburg 35037, Germany; Department of Pharmacy, Institute of Pharmaceutical Chemistry, Marburg University, Marburg 35037, Germany; Department of Pharmacy, Institute of Pharmaceutical Chemistry, Marburg University, Marburg 35037, Germany; Institute for Organic Chemistry and Chemical Biology, Center for Biomolecular Magnetic Resonance (BMRZ), Goethe University Frankfurt am Main, Frankfurt/Main 60438, Germany; Department of Pharmacy, Institute of Pharmaceutical Chemistry, Marburg University, Marburg 35037, Germany; Institute for Organic Chemistry and Chemical Biology, Center for Biomolecular Magnetic Resonance (BMRZ), Goethe University Frankfurt am Main, Frankfurt/Main 60438, Germany; Institute for Organic Chemistry and Chemical Biology, Center for Biomolecular Magnetic Resonance (BMRZ), Goethe University Frankfurt am Main, Frankfurt/Main 60438, Germany; Department of Pharmacy, Institute of Pharmaceutical Chemistry, Marburg University, Marburg 35037, Germany; Center for Synthetic Microbiology (SYNMIKRO), Marburg University, Marburg 35043, Germany

## Abstract

Several SARS-CoV-2 variants have evolved with clinical relevance due to their structural and functional impact on viral proteins and genomic RNA structures. A comprehensive structural and functional characterization of single-nucleotide variations in the 5′-untranslated region (UTR) of SARS-CoV-2 has remained elusive. The co-evolution of 5′-UTR stem-loop 1 (SL1) and non-structural protein (nsp) 1 mutants are of particular importance, as both are key in directing the translation of the viral genome. Here, we investigate the structure and function of the most frequently emerging mutations in SL1 and nsp1. Mutation C21U in the apical loop of SL1 shows changed loop dynamics and reduced escape from repression by nsp1 mutants. Mutation analyses of the pyrimidine loop and the apical helix of SL1 identify preferred sequence motifs for escape from nsp1 repression. Importantly, sequence preferences are governed by the structural features, with suboptimal pyrimidine sequences escaping repression when presented in the SL1 context. Compared to the nsp1 wild type (wt), the currently circulating nsp1 linker variant S135R is much more sensitive toward sequence and structure variations of the apical loop of SL1. Thus, our study provides new insights into the structure–function relationship and co-evolution between viral RNA structures and viral proteins.

## Graphical Abstract

**Graphical Abstract:**
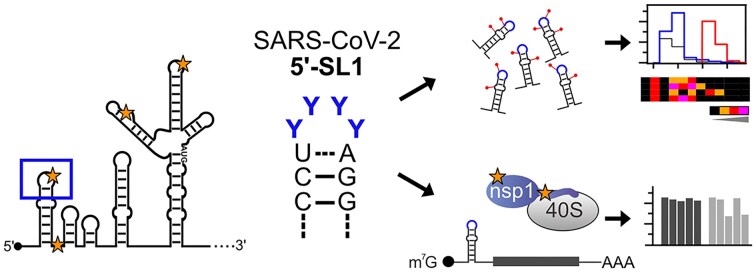

## Introduction

The highly structured single-stranded positive-sense RNA genome of SARS-CoV-2 comprises ~30 kilobases (kb). It encodes a set of non-structural proteins (nsps 1–16) as well as the structural proteins spike (S), envelope (E), membrane (M), nucleocapsid (N), and several accessory factors [1, 2]. The functional interaction of these viral proteins with the coronaviral genome involves highly conserved and essential *cis*-regulatory RNA elements in coding and non-coding regions. Several of these RNA structures are located in the 5′- and 3′-untranslated region (UTR) [3–5]. Accordingly, the 5′-UTR of SARS-CoV-2 is highly structured as shown by structural probing *in vitro, in virion*, and in virus-infected cells [6–11]. Five SLs (SL1–SL5) have been identified in the 5′-UTR that were also shown to fold independently as isolated RNA structures *in vitro* [6], with SL5 containing the canonical translation start codon. While it has been shown that all five SLs are essential for the viral life cycle, a detailed understanding of their structure–function relationship and interaction with viral proteins is mostly missing [3, 5].

Nsp1 is unique for coronaviruses and supports hijacking of the host cell by redirecting translation towards viral RNAs. SARS-CoV-2 nsp1 comprises 180 amino acids. It consists of an unstructured N-terminal tail, a globular N-terminal domain (NTD) as well as highly disordered linker region and C-terminal domain (CTD) [12]. Nsp1 binds to the ribosomal 40S subunit by insertion of its CTD into the messenger RNA (mRNA) entry channel. There, it is in contact with ribosomal proteins (uS3, uS5, and eS30) as well as ribosomal RNA (18S) and blocks mRNA entry [13–15]. In consequence, nsp1 globally inhibits translation of host mRNAs [15–17]. In this, the conserved residues K164 and H165 were shown to be essential for ribosome binding and repression of translation [14]. Further, nsp1 also induces mRNA degradation, which was shown to be dependent on translation initiation and binding of nsp1 to the ribosomal 40S subunit [18–21].

Intriguingly, the presence of SL1 alone, as an isolated RNA element at their 5′-end, is sufficient for (viral) mRNAs to selectively escape from nsp1 repression [22–24]. SL1 encompasses the nucleotides (nts) 7–33 and comprises two helical segments (G7-C33 to U11-A29 and U13-A26 to U17-A22), an asymmetrical internal loop (A12; A27 and C28), and an apical tetraloop consisting of four pyrimidines U18, C19, C20, and C21 with a labile closing base pair U17-A22 [25, 26]. A guanosine-free 5′-proximal sequence stretch in SL1 (nts 9–22) seems to be necessary to mediate escape from nsp1 [27]. Accordingly, mutation of C19 and C20 in the apical tetraloop to guanosines is detrimental to escape [24, 27, 28]. However, how SL1 is specifically recognized by nsp1 is not clear, with the majority of studies reporting no RNA binding of free full-length nsp1 *in vitro* [12, 23, 29, 30]. Nevertheless, co-immunoprecipitation experiments with SARS-CoV-2 nsp1 show enrichment of mRNA only when SL1 is present [22, 29]. In this, the NTD is essential for RNA co-immunoprecipitation. Especially amino acids R124 and K125 have been shown to be important for co-immunoprecipitation of SL1-containing transcripts [22]. Strikingly, SL1 and nsp1 co-evolve and are specific for each coronavirus [31].

Since the outbreak of the SARS-CoV-2 pandemic, a large number of mutations of the viral genome have been reported [32]. Several of these viral variants raised concern of increased virulence, transmissibility, and antigenicity of the virus [33]. For nsp1, mutation K47R located in the NTD and mutation S135R as part of the linker region have been described, but were not functionally investigated so far [32]. Interestingly, also mutations in the non-coding UTRs were reported. However, to date, a comprehensive combined structural and functional characterization of the mutations in the 5′-UTR of SARS-CoV-2 is missing, yet necessary to gain a better understanding of the relevance and function of its RNA elements. Previously, we characterized the impact on local RNA structure and dynamics of the two most frequent mutations in SL5 (G210U in SL5a and C241U in SL5b) by nuclear magnetic resonance (NMR) spectroscopy. SL5 comprises the nts 150–294. It is folded into a complex four-way junction with a closing stem and three sub-elements SL5a, 5b, and 5c, which together adopt a T-shaped three-dimensional tertiary structure as shown by cryo-EM [34, 35]. Mutation C241U was shown to destabilize the SL5b hexaloop, while mutation G210U alters the base-pairing pattern in the asymmetrical bulge of SL5a [36].

To gain further insight into how the 5′-UTR mutations affect RNA folding in the full 5′-UTR context, we perform structural probing using *in vitro* SHAPE-MaP (selective 2′-hydroxyl acylation analyzed by primer extension and mutational profiling). We focus on the four most frequent mutations that occurred in different combinations in SARS-CoV-2 variants (C21U, C44U, G210U, and C241). All mutations preserve the overall 5′-UTR secondary structure, but change local structural dynamics.

Starting from mutation C21U in the apical loop of SL1, we perform a detailed analysis of the structure–function relationship of this *cis*-regulatory RNA. We demonstrate that loop dynamics and the pyrimidine sequence of SL1 together modulate the escape from nsp1-mediated repression. The nsp1 wt and the currently circulating nsp1 variant S135R show differential robustness against sequence and structural changes in SL1. Thus, our integrative structural and functional analysis of viral mutants provides new details on key characteristics in the SL1/nsp1 interplay.

## Materials and methods

### Plasmid construction

*In vitro-transcription (IVT) templates for SHAPE-MaP and CD-spectroscopy*. The 5′-UTR wt sequence of SARS-CoV-2 (NC_045 512) was polymerase chain reaction (PCR) amplified from pSP64_SCoV2_5′-UTR [6] and inserted into plasmid pCR2.1_TOPO_T7HHtRNAPheHDV encoding sequence-optimized hammerhead (HH) and human delta virus (HDV) ribozymes [37, 38], resulting in the final vector pCR2.1TOPO_T7-HH(adapted)_5′UTR_CoV19_wt-HDV. Point mutants of variants of interest according to publicly available GISAID data on www.nextstrain.org [32] were introduced by site-directed mutagenesis PCR (Supplementary Table S1).

For constructs shuffling the pyrimidine bases of the SARS-CoV-2 SL1 apical loop (SL1_YYYY) the 5′-UTR was shortened to include only SL1 and SL2, mutating the four pyrimidine bases to all 16 possible combinations, while introducing an additional barcode stem at the 3′-end to barcode each construct [39] by mutagenesis PCR (Supplementary Table S2). Additionally, closing base pair mutants U17C_A22G, A22G, and U17A_A22U were prepared the same way. Barcode sequences had a minimum of three Levenshtein distances, and the structure of the complete RNA constructs was modeled with RNAstructure web servers (https://rna.urmc.rochester.edu/RNAstructureWeb/) [40] to ensure native-like folding of all stems.

pHDV-5_SL1CoV2wt was prepared by hybridizing and phosphorylating two complementary DNA oligonucleotides carrying the nucleotides 7–33 of SARS-CoV-2 SL1 and subsequent cloning into pHDV [41] via restriction sites *Nco*I and *Hind*III. SL1 constructs for circular dichroism (CD) experiments were created by site-directed mutagenesis PCR of pHDV-5_SL1CoV2wt (see Table 1).

**Table 1.** Melting points, respective errors and sequences of the selected SL1 pyrimidine mutants

Tetraloopsequence	Meltingpoint (°C)	Sequence
UCCC (wt)	66.5 ± 0.5	5'-GGUUUAUACCU **UCCC** AGGUAACAAACC-3'
CCCC	68.1 ± 0.6	5'-GGUUUAUACCU **CCCC** AGGUAACAAACC-3'
CUUU	65.3 ± 0.2	5'-GGUUUAUACCU **CUUU** AGGUAACAAACC-3'
UCCU (C21U)	64.7 ± 0.3	5'-GGUUUAUACCU **UCCU** AGGUAACAAACC-3'
UCUU	65.6 ± 0.3	5'-GGUUUAUACCU **UCUU** AGGUAACAAACC-3'

Apical loop sequences are bold.

*SARS-CoV-2 and SARS-CoV nsp1 plasmids*. The coding sequences of SARS-CoV-2 wt nsp1 (NC_045 512) and SARS-CoV wt nsp1 (NC_004 718) were ordered at GeneArt™ Strings™ DNA Fragments (Invitrogen) and amplified with PCR. The amplified inserts were cloned into pCMV-MS [42] via restriction sites *Xho*I and *Mlu*I, resulting in the final plasmids pCMV_SARS-CoV-2_nsp1_wt and pCMV_SARS-CoV_nsp1_wt. SARS-CoV-2 and SARS-CoV nsp1 variant nsp1_S135R and SARS-CoV-2 variant nsp1_K47R + S135R were cloned by site-directed mutagenesis PCR of the respective wt plasmids.

*Dual luciferase reporter plasmids*. We created two Golden Gate pDL_GG plasmid variants out of the original pDL (hRluc/fLuc) plasmid [42] by introducing *Bsa*I restriction sites in the 5′-UTR upstream of the firefly luciferase (fLuc) ORF by site-directed mutagenesis PCR. In pDL_GG_5UTR_fLuc_noATG, the start codon of firefly luciferase was removed to allow for seamless introduction of full-length SARS-CoV-2 5′-UTR (Supplementary Table S3). SARS-CoV-2 5′-UTR variants were PCR-amplified from the transcription cassettes introducing *BsaI* restriction sites. PCR products were subsequently cloned into pDL_GG_5UTR_fLuc_noATG by Golden Gate cloning. For the plasmid pDL_GG_5UTR_fLuc_+ATG the start codon was maintained allowing the introduction of SL1 constructs. SL1 constructs were introduced with Golden Gate cloning using hybridized and phosphorylated DNA oligonucleotides (Supplementary Table S4).

*Single firefly luciferase reporter plasmids*. The CMV promoter/enhancer element was amplified from pDLP [42], introducing the SARS-CoV-2 SL1 wt into the 5′-UTR (Supplementary Table S4) by PCR. The PCR product was cloned into pGL4.10 (Promega) containing the firefly luciferase gene (fLuc) via *Sac*I and *Apa*I restriction sites, resulting in the final vector pGL_SCoV2_5_SL1_wt. Using this plasmid, we introduced *Bsa*I restriction sites in the 5′-UTR upstream of the firefly luciferase ORF by site-directed mutagenesis PCR, resulting in the final plasmid pGL_GG_5UTR_fLuc. SL1 constructs (Supplementary Table S4) were either cloned by site-directed mutagenesis of the vector pGL_SCoV2_5_SL1_wt or by Golden Gate cloning using hybridized and phosphorylated DNA oligonucleotides pGL_GG_5UTR_fLuc.

All plasmid and oligonucleotide sequences are available upon request.

### RNA preparation and purification

IVT templates for SHAPE-MaP of full-length 5′-UTRs, barcoded pyrimidine constructs (SL1_YYYY), and closing base pair mutants were generated by PCR from the above-described plasmids using the oligonucleotides forward: 5′-GGAGATCTAATACGACTCAC-3′ and reverse: 5′-AAACGACGGCCAGTGC-3′. DNA from PCR reactions was purified using the DNA Clean & Concentrator kit (Zymo Research).

For SL1 CD experiments, plasmids were linearized with HindIII (New England Biolabs). Linearized plasmids were purified by phenol-chloroform extraction followed by precipitation with 2-propanol. Linearized and purified plasmid DNA was used as IVT template.

All RNAs were prepared using in-house purified T7 RNA-polymerase with 200 mM Tris–HCl (pH 8.0), 20 mM dithiothreitol (DTT), 2mM spermidine, and 4 mM NTP (each). For full-length 5′-UTRs, barcoded pyrimidine constructs (SL1_YYYY) and closing base pair mutants, dimethyl sulfoxide (DMSO) was added to a final concentration of 5% (v/v) and magnesium acetate Mg(Ac)_2_ to a final concentration of 30 mM. Barcoded constructs were *in vitro* transcribed individually and pooled equimolarly afterward. For SL1 CD constructs, DMSO and magnesium acetate concentrations were 10% and 20 mM, respectively.

All RNAs were *in vitro* transcribed at 37°C for 2 h or overnight and subsequently purified by Urea–polyacrylamide gel electrophoresis (PAGE) as described previously [6].

### SHAPE-MaP

SHAPE-MaP was performed as described previously [43, 44]. Briefly, RNA folded by snap-cooling was probed at a final concentration of 0.5 mM RNA and 5 mM 5-nitroisatoic anhydride (5-NIA) at 37°C for 15 min in 1× folding buffer (100 mM HEPES, pH 8.0, 100 mM NaCl, and 10 mM MgCl_2_). Additionally, DMSO control and denaturing control were performed as described previously [44]. After purification with RNA Clean and Concentrator (Zymo Research), preadenylated adapter (5′-P-(rA)GATCGGAAGAGCACACGTCTGAACTCCAG TC-Amino-C6-3′) was ligated at the 3′-end, followed by reverse transcription with a circularization primer (5′-AGATCGGAAGAGCGTCGTGTAGGGAAAGAGTGT-iSp18-CACTCA-iSp18-GACTGGAGTTCAGACGTGTGCTCTTCCGATCT-3′). cDNA was digested with RNase H (Thermo Fisher Scientific) for 30 min at 37°C and purified with the RNA Clean & Concentrator kit (Zymo Research). Circularization was done by T4 RNA ligase (New England Biolabs) and libraries barcoded with unique dual indices. NGS of the libraries was performed at GENEWIZ (Azenta, Leipzig, Germany) on an Illumina NovaSeq system, and data was delivered via SFTP. For the pool of SL1_YYYY constructs, 575 million paired-end reads (2 × 150 bp) were received. For the full-length 5′-UTR constructs, between 40 and 100 million paired-end reads (2 × 150 bp) were received. Quality control of the NGS data was performed with FastQC v0.12.1 (https://github.com/s-andrews/FastQC) and NGS data was used for analysis with Shapemapper2 v2.2 (https://github.com/Weeks-UNC/shapemapper2)[45].

SHAPE reactivities for full-length 5′-UTRs were calculated using the box-plot normalization scheme described previously [46]. Briefly, a normalization factor was determined by calculation of the interquartile range (IQR), and values above 1.5 times the IQR were removed. The next 10% highest reactivities were averaged, and all reactivities were divided by this normalization factor to yield SHAPE reactivities. Data were plotted with either ggplot2 (step-plots, [47]) or matplotlib (heatmap, [48]) and identical colors to SHAPE-reactivity visualization by the Shapemapper2 pipeline were chosen, expanded to include visualization of hyper-reactive nucleotides [49]. Due to unusually high mutation rates for some nucleotides in the untreated and denatured samples for the SARS-CoV-2 5′-UTR C21U_C44U_C241U mutant, the mutation rates for this mutant were recalculated by averaging over the untreated mutation rates of all full-length 5′-UTRs for untreated and denatured samples.

SHAPE reactivities for barcoded SL1-2 constructs (SL1_YYYY) and closing base pair mutants were derived from Shapemapper2 v2.2 output.

Secondary structure prediction with SHAPE reactivity constraints was performed with RNAstructure web servers [40]. RNAcanvas was used for the graphical representation of RNA structures [50].

### Circular dichroism spectroscopy and melting point determination

SL1 RNA constructs were refolded via snap-cooling prior to measurements by incubation at 95°C for 30 s and subsequent storage on ice for 5 min. SL1 constructs were measured in 1× folding buffer in a 1-mm cuvette with a final RNA concentration of 10 μM. CD experiments were conducted using a Jasco J-810 spectropolarimeter in the spectral range of 320–220 nm at 25°C and 37°C. The CD spectra were recorded with a scanning speed of 50 nm/min and a data interval of 0.5 nm. All spectra were further processed by applying a Savitzky–Golay smoothing filter with 15 points. The measured ellipticities were converted from milidegree (mdeg) to molar ellipticity [θ] = deg × cm^2^ × dmol^−1^.

Melting curves were measured at a constant wavelength corresponding to the maximum CD signal observed in the spectrum recorded at 25°C (wt = 269.5 nm, CCCC = 272 nm, CUUU = 268.5 nm, UCCU = 265.5 nm, UCUU = 268 nm). The CD signal was recorded in a temperature interval from 10°C to 90°C with a sampling rate of 0.5°C/min. The recorded data points were normalized to the ten highest and lowest CD values, then fitted with equation 1 using OriginPro^®^ 2023 to determine the melting points. A1 represents the lowest and A2 the highest normalized ellipticity, LOG_x0_ the melting point, and p the proportion.


(1) \begin{eqnarray*}
y = A1 + \frac{{\left( {A2 - A1} \right)}}{{\left( {1 + \ {{{10}}^{\left( {\left( {LO{{G}_{x0}} - x} \right)*p} \right)}}} \right)}}.
\end{eqnarray*}

### Nuclear magnetic resonance spectroscopy

NMR experiments were conducted using natural abundance (n.a.) RNA samples, obtained either through in-house IVT or purchased from Horizon Discovery. The sequences of the RNA constructs used, SL1 wt and SL1 C21U, contained the nucleotides 7 to 33 of the SARS-CoV-2 RNA genome (Supplementary Table S5). These sequences were further stabilized by an additional G-C base pair at the terminal end. All RNAs were purified by reverse-phase high-performance liquid chromatography (HPLC) on a Kromasil RP-18 column applying a 0%–40% gradient of 0.1 M acetonitrile/triethylammonium acetate. RNA-containing fractions were freeze-dried, followed by cation exchange via LiClO_4_ precipitation [2% (w/v) in acetone]. The RNA was then folded by heating to 80°C in water and rapidly cooling on ice. Subsequent buffer exchange into NMR buffer (25 mM potassium phosphate, pH 6.2, 50 mM potassium chloride) was performed using 2 kDa molecular weight cut-off (MWCO) centrifugal concentrators. Sample purity was confirmed by denaturing PAGE, and homogenous folding was validated by native PAGE, using the same RNA concentrations as applied in the NMR measurements. The NMR measurements were conducted as described previously [25, 26]. Briefly, NMR spectra were acquired at 900 MHz and 278 K. For comparison of SL1 wt and SL1 C21U, 2D-^1^H,^15^N-Heteronuclear single quantum coherence spectroscopy (HSQC) [51] and 2D-^1^H,^1^H-Nuclear Overhauser effect and Exchange spectroscopy (NOESY; mixing time: 100 ms) [52, 53] experiments were performed, and spectral overlays were analyzed to assess structural differences.

### Cell culture and transfection

HEK293 cells (DSMZ, Germany, ACC305) were cultured in Dulbecco’s modified Eagle’s medium (Gibco) supplemented with 10% fetal bovine serum (FBS superior, Sigma–Aldrich), 1% sodium pyruvate (Gibco), and Penicillin–Streptomycin (Gibco) at 37°C and 5% CO_2_ in a humidified incubator.

For transfections of reporter plasmids for luciferase experiments, HEK293 cells were seeded in 96-well plates with 20 000 cells/well and incubated at 37°C and 5% CO_2_. After 24 h, cells were transfected using Lipofectamine™ 2000 (Thermo Fisher Scientific) according to the manufacturer’s protocol. For dual luciferase experiments without co-expressing SARS-CoV-2 nsp1 or eGFP, a total of 10 ng pDL (hRluc/fLuc) reporter plasmid was transfected per well. For co-expression experiments using the dual luciferase reporter, 2.5 ng of pDL (hRluc/fLuc) reporter plasmid and 17.5 ng of either nsp1 plasmid (wt or mutants) or eGFP plasmid (control) were transfected per well. For single luciferase experiments, 5 ng of pGL reporter plasmids were co-transfected with 10 ng of either nsp1 plasmids (wt or mutants) or eGFP plasmid (control) per well.

For western blot experiments, HEK293 cells were seeded in 12-well plates with 240 000 cells/well and incubated at 37°C and 5% CO_2_. After 24 h, cells were transfected with 110 ng (single luciferase constructs) or 191.5 ng (dual luciferase constructs) of the respective nsp1 or eGFP plasmids using Lipofectamine™ 2000 (Thermo Fisher Scientific) according to the manufacturer’s protocol.

For total RNA extraction for RT-qPCR experiments, HEK293 cells were seeded in 12-well plates with 240 000 cells/well and incubated at 37°C and 5% CO_2_. For dual luciferase constructs with co-expression of eGFP (control) or nsp1 (wt and mutants), 27.5 ng of pDL (hRluc/fluc) reporter plasmid and 191.5 ng of either nsp1 or eGFP plasmids were co-transfected per well using Lipofectamine™ 2000 (Thermo Fisher Scientific) according to the manufacturer’s protocol. For single luciferase constructs, 55 ng of respective pGL reporter plasmids were co-transfected with 110 ng of either nsp1 plasmids (wt or mutant S135R) or eGFP plasmid (control) per well using Lipofectamine™ 2000 (Thermo Fisher Scientific) according to the manufacturer’s protocol.

### Luciferase reporter assays

Luciferase reporter assays were performed 24 h after transfection. All luminescence measurements were performed with a TECAN Infinite M Plex plate reader (TECAN, Switzerland). The dual luciferase reporter assay system (Promega, cat. no. E2940) was used according to the manufacturer’s protocol (pDL plasmids). All replicates were performed in technical triplicates. Data evaluation was performed by normalizing the firefly luciferase activity to *Renilla* luciferase activity serving as internal control according to equation 2 for experiments without co-transfection of nsp1 or eGFP:


(2) \begin{eqnarray*}
\mathrm{ratio}\ \mathrm{fLuc}/hRluc = \frac{{RLU\ \left( {\mathrm{fLuc}} \right)}}{{RLU\ \left( {\mathrm{hRluc}} \right)}}.
\end{eqnarray*}

For dual luciferase experiments with co-transfection of nsp1 or eGFP plasmids, the normalized luciferase activity was calculated according to the following equation (3):


(3) \begin{eqnarray*}
\mathrm{norm}.\ \mathrm{ratio}\ \mathrm{fLuc}/hRluc\ = \frac{{{{{\left( {\mathrm{ratio}\ \mathrm{fLuc}/hRluc} \right)}}_{nsp1}}}}{{{{{(\mathrm{ratio}\ \mathrm{fLuc}/hRluc)}}_{\mathrm{eGFP}}}}}.
\end{eqnarray*}

Luciferase reporter assays using the firefly luciferase as single reporter (pGL plasmids) were performed using the ONE-Glo™ EX Luciferase Assay System (Promega, cat. no. E8120) according to the manufacturer’s protocol. All measurements were performed in technical triplicates. Data evaluation was performed by normalizing the firefly luciferase activity in co-expression with nsp1 (variants) to the corresponding luciferase activity in co-expression with eGFP according to equation (4):


(4) \begin{eqnarray*}
\mathrm{norm}.\ \mathrm{fLuc}\ \mathrm{activity}\ = \frac{{RL{{U}_{nsp1}}}}{{RL{{U}_{\mathrm{eGFP}}}}}.
\end{eqnarray*}

### Western blot

For western blot analysis of nsp1 levels, cells were washed with pre-warmed 1× PBS 24 h after transfection and then lysed with lysis buffer [137.5 mM NaCl, 10% glycerol, 20 mM Tris–HCl, pH 8.0, 2 mM EDTA, 1% Igepal, 0.005% Protease Inhibitor Cocktail (Sigma–Aldrich)] for 20 min on ice. Lysates were transferred into reaction tubes and centrifuged for 15 min at 17 000 × *g* and 4°C. After centrifugation, the protein concentration of the supernatants was determined according to the Bradford method in technical triplicates. Subsequently, 10 µg of total protein of each sample was loaded onto a 15% SDS–PAGE containing trichloroethanol (TCE) for staining of total protein. Total lane protein was visualized using the ChemiDoc Imaging System (Bio-Rad). Afterward, proteins were blotted onto a PVDF membrane according to the manufacturer’s instructions (Bio-Rad). Detection of SARS-CoV-2 nsp1 was performed using a target-specific SARS-CoV-2 (COVID-19) nsp1 primary antibody (GeneTex, GTX135612) (1:1000 in 5% skimmed milk in 1× TBS-T; incubation: 30 min) and horseradish peroxidase-conjugated anti-rabbit IgG (Jackson Immunoresearch) as secondary antibody. For visualization, the WesternBright ECL HRP substrate (Advansta Inc.) was used according to the manufacturer’s protocol. Images were recorded with the ChemiDoc Imaging System (Bio-Rad) and analyzed and quantified using the Image Lab software v6.1 (Bio-Rad).

### RNA isolation and RT-qPCR

Total RNA was extracted from HEK293 cells 24 h after transfection. Cells were washed with pre-warmed 1× PBS followed by lysis with TRIzol™ reagent (Thermo Fisher Scientific). Phase separation was performed according to the manufacturer’s instructions. The aqueous phase was transferred into a fresh tube and mixed with one sample volume of 100% ethanol. Mixed samples were purified using the RNA Clean & Concentrator kit (Zymo Research) according to the manufacturer’s protocol. After purification, extracted total RNA was treated with Turbo™ DNase (Thermo Fisher Scientific) for 45 min at 37°C. Subsequently, digested samples were purified using the RNA Clean & Concentrator kit (Zymo Research). Purified total RNA was checked for quality and integrity on a 1% agarose gel. For analysis of mRNA levels, 1 µg of total RNA was reverse transcribed with random hexamers (Jena Bioscience) and GoScript™ Reverse Transcriptase (Promega). cDNA was quantified by qPCR using the QuantStudio 3 Real-Time-PCR system (Thermo Fisher Scientific) and Fast SYBR^®^ Green Master Mix (Thermo Fisher Scientific) with target specific primers (fLuc: forward: 5′-GCTCAGCAAGGAGGTAGGTG-3′, reverse: 5′-TCTTACCGGTGTCCAAGTCC-3′ [42]; hRLuc: 5′-CTAACCTCGCCCTTCTCCTT-3′, reverse: 5′-TCGTCCATGCTGAGAGTGTC-3′ [42]; 18S: 5′-GTAACCCGTTGAACCCCATT-3′, reverse: 5′-CCATCCAATCGGTAGTAGCG-3′ [29]). Firefly mRNA levels were calculated relative to the respective reference gene (=2^−ΔCt^), *Renilla* luciferase for dual luciferase constructs and 18S rRNA for single luciferase constructs. Relative mRNA levels were determined by normalizing the relative firefly mRNA levels with nsp1 wt, the single mutant S135R or the double mutant K47R/S135R co-transfection to the relative firefly mRNA levels of the respective construct with eGFP co-transfection.

## Results

### Nucleotide changes in the 5′-UTR of SARS-CoV-2 variants alter local RNA dynamics

**Figure 1.**
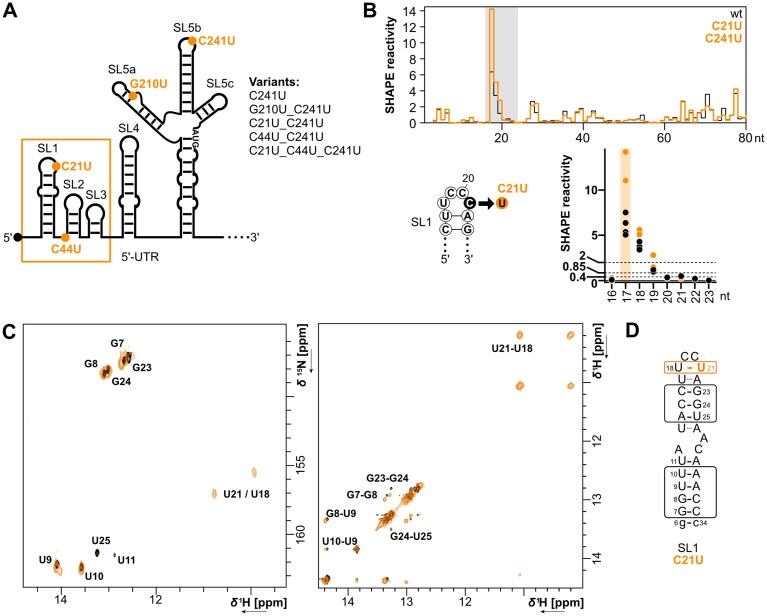
Nucleotide changes in the 5′-UTR of SARS-CoV-2 variants alter local structural dynamics. (**A**) Schematic representation of the secondary structure of the SARS-CoV-2 5′-UTR and position of mutations in variants. (**B**) Top: SHAPE reactivity comparison of probed mutations C21U/C241U (orange line) and the wt (black line). Area of interest is highlighted in gray. Bottom: Comparison of SHAPE reactivities of the area of interest of tested SARS-CoV-2 variant 5′-UTRs with (orange dots) or without (black dots) mutation C21U. (**C**) Comparison between SL1 wt (black) and SL C21U (orange) reveals an additional base pair in the apical loop for the mutant (left). 2D-1H,15N-HSQC overlays of SL1 wt and SL1 C21U. 2D-1H,1H-NOESY overlays of SL1 wt and C21U (mixing time = 100 ms) (right). NMR data were acquired at pH 6.2, 900 MHz and 278 K. (**D**) Schematic representation of the secondary structure of SL1 C21U as determined by NMR.

To characterize, whether evolving nucleotide changes in SL1–SL5 at the 5′-end of the SARS-CoV-2 genome (UTR: nt 1–265; coding region of nsp1: nt 266–294) affect RNA secondary structure, we performed *in vitro* SHAPE-MaP of the wt and the most frequent variants (C241U; G210U/C241U; C44U/C241U; C21U/C241U; C21U/C44U/C241U) (Fig. 1A). SHAPE-MaP data of the SARS-CoV-2 5′-UTR wt (Supplementary Fig. S1A and Supplementary Table S6) were in perfect agreement with previously determined secondary structure models *in vitro* and from virus-infected cells [6, 7, 8]. Comparison of SHAPE reactivity profiles of mutant variants with the wt (Supplementary Fig. S1B and Supplementary Fig. S2) showed that overall folding of the SARS-CoV-2 5′-UTR was preserved. However, some mutations strongly affected nucleotide reactivities in their close proximity, thus demonstrating their impact on local RNA structural dynamics.

Interestingly, comparison of SHAPE-MaP data from mutants to wt show several consistent changes in hyperreactivity of nucleotides (SHAPE reactivity >2). Hyper-reactive nucleotides are either highly flexible or adopt a constrained conformation, which activates the 2′-OH group [49, 54]. The reactivity of the 2′-OH group in such constrained, hyper-reactive nucleotides originates from nucleotide conformations where the non-bridging oxygen atoms from the adjacent 3′-phosphodiester are pointed away from the 2′-OH group or by deprotonation of the 2′-OH group by functional proximal groups through general base catalysis [49]. For the latter mechanism, interaction of the 2′-OH with the O2 of the attached pyrimidine base appeared to be particularly favored. Additionally, the reactivity of the 2′-OH group can be affected by neighboring nucleotides and through-space interactions to bring the 2′-OH close to a general base group [49]. Thus, mutations might not only affect the SHAPE reactivity of the mutated nucleotide itself, but lead to larger changes in neighboring nucleotides indicative of structural rearrangements.

C241U initially occurred at the beginning of the global pandemic in 2020, dominating all following variants to date (Supplementary Fig. S3). The mutation is located in the hexaloop of sub-element SL5b changing it from UUUCGU to UUUUGU. Variants with mutation C241U showed a decisive drop of SHAPE reactivity at position U238, turning it non-reactive in comparison to the hyper-reactive U238 in the wt (Supplementary Fig. S2B). In addition, SHAPE reactivity slightly increased for positions U239 to G242 (Supplementary Fig. S2B). Consequently, these multiple changes imply a local conformational re-arrangement of the apical loop of SL5b in response to mutation C241U and are consistent with NMR experiments showing a destabilization of the mutated SL5b hexaloop [36]. SL5 mutant G210U/C241U was exclusively predominant during the rise of the 21A Delta variant by the second half of 2021 (Supplementary Fig. S3). G210U is located in the asymmetrical internal loop of SL5a. For this variant, an increase of SHAPE reactivity was observed at the mutated position 210, becoming hyper-reactive compared to the wt and variants without the respective mutation (Supplementary Fig. S2C). This increase in reactivity is consistent with a changed base-pairing pattern in the asymmetrical loop observed by NMR. There, G210 is involved in base-pairing, while U210 in not. Furthermore, position U194 on the opposing strand of the asymmetric loop turned non-reactive exclusively in variant G210U/C241U (Supplementary Fig. S2C). This is in line with the stabilization of the U194–U212 base pair in the G210U mutant, also observed by NMR [36, 55]. Thus, SHAPE data of both mutations in full-length SL5 are consistent with previous detailed structural analyses of the isolated sub-elements with NMR, emphasizing the strength of SHAPE-MaP to dissect changes in local RNA dynamics upon single nucleotide mutations.

We next focused on reactivity changes upon mutation C21U, to gain a deeper understanding of the structure-function relationship in SL1 important to evade repression from nsp1. C21U was described in the first emerging Omicron variant BA.1 by the end of 2021 (Supplementary Fig. S3). Subsequently, C21U disappeared by mid-2022 and reappeared again at the beginning of 2024 in JN.1 variants (Supplementary Fig. S3). SL1 folds into a bipartite SL structure with an apical UCCC tetraloop (nts 18–21), closed by a labile U-A base pair [6, 25]. Comparing the SHAPE reactivity profiles of the tested SARS-CoV-2 5′-UTRs variants, overall SHAPE reactivity was high for nts U17 of the closing base pair and U18/C19 of the apical loop (Fig. 1B and Supplementary Fig. S1B). This asymmetry of higher SHAPE reactivity of nucleotides at the 5′-end of apical loops has been noted before and is most striking for the closing base pair with the 5′-nucleotide being highly reactive, while the 3′-nucleotide is not [56]. Additionally, reactivity profiles of all variants confirm the presence of the asymmetric internal loop (Supplementary Fig. S1B). The reactivity profiles are consistent with highly dynamic apical and internal loops in accordance with previous observations by NMR [25, 26]. Interestingly, mutation C21U caused an ~2-fold increase in SHAPE hyper-reactivity at position U17. Thus, the nucleotide identity at position four in the SL1 apical loop (nt 21) affects the reactivity of the nucleotide preceding the loop (nt 17). In consequence, the increase of hyper-reactivity of U17 results from either higher flexibility and thus a more labile closing base-pair or conformational changes, which constrain the nucleotide in a highly reactive conformation.

To characterize this change in hyper-reactivity in more detail, we compared the structure of the SL1 wt to the reported variant C21U by measuring samples of both RNAs using NMR spectroscopy. Chemical shift differences between SL1 wt and C21U observed in the 2D-^1^H,^15^N-HSQC together with NOE cross-peaks in the 2D-^1^H,^1^H-NOESY spectrum (Fig. 1C) are consistent with the formation of a non-canonical U•U interaction between U21 and U18 (Fig. 1D). Such U•U wobble base pairs perturb the A-helix geometry, also of their adjacent base pairs [57]. In the case of C21U, such a perturbation of the labile base pair U17-A22 [25] could result in the increased hyper-reactivity observed at U17 as observed in SHAPE experiments.

In sum, SHAPE-MaP shows changes in local RNA dynamics of structural RNA elements in the variant SARS-CoV-2 5′-UTRs which are supported by NMR. Particularly, changes in RNA dynamics caused by the C21U mutation in SL1 are of high functional interest for the translation of viral RNAs.

### SL1 mutation C21U affects escape from nsp1-mediated repression

To elucidate the functional impact on the translation efficiency of SARS-CoV-2 variants, we performed dual luciferase reporter gene assays in HEK293 cells of respective 5′-UTR variants (Supplementary Fig. S2D). Here, firefly luciferase is used as reporter, while *Renilla* luciferase serves as intrinsic control. We observed only minor alterations in the range of ~10% for the translation efficiency for all SARS-CoV-2 variants, including variants with mutation C21U in comparison to the wt. Hence, the local changes in RNA dynamics do not affect the basic translation efficiency of the SARS-CoV-2 variants. However, nsp1 modulates the translation efficiency of viral mRNAs in an infection context. Thus, we next investigated the interplay of SL1 RNA and nsp1 protein variants.

**Figure 2.**
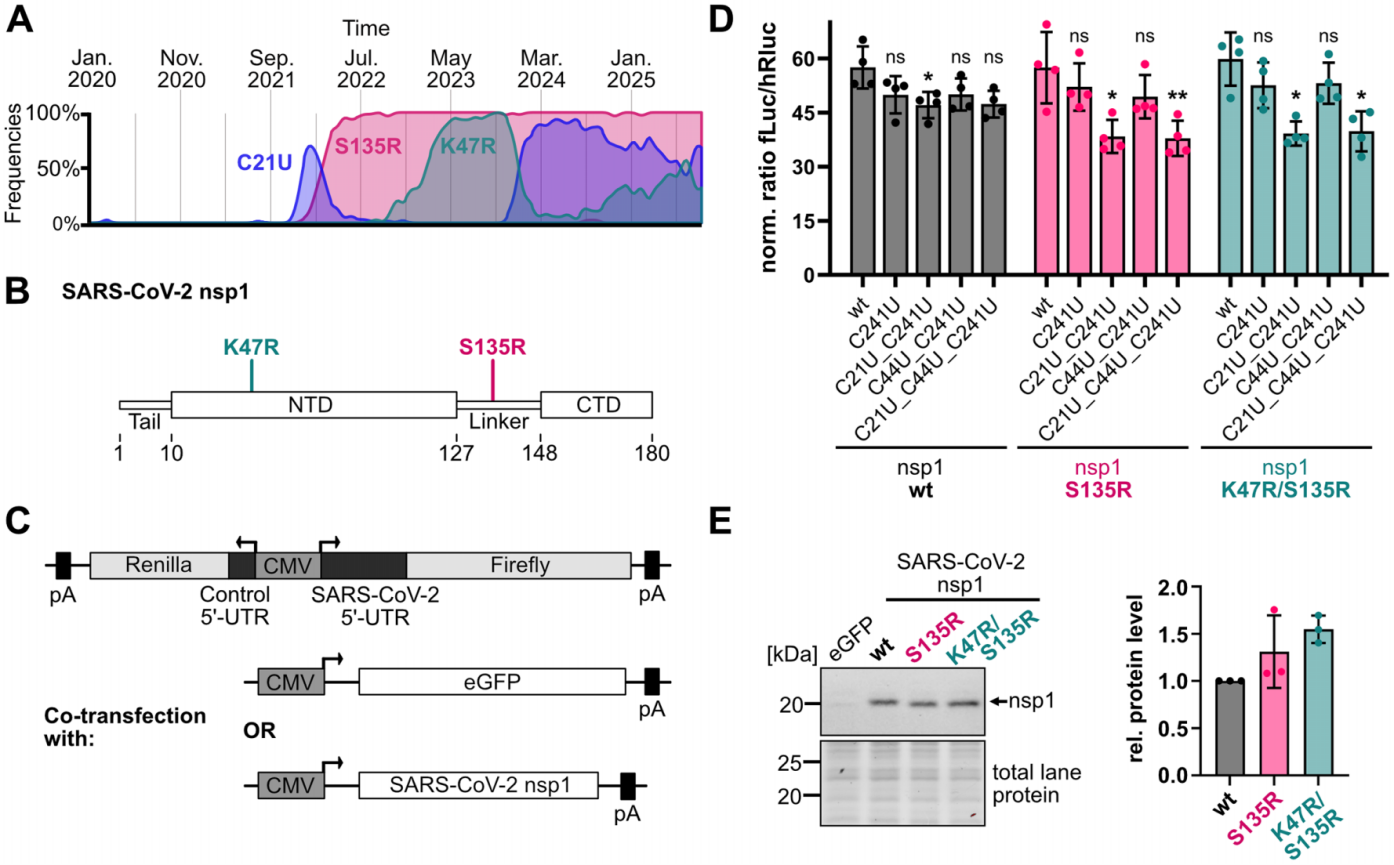
SL1 mutation C21U affects escape from nsp1-mediated repression. (**A**) Time course of the occurrence of mutation C21U and mutations K47R (A405G) and S135R (U670G) in the coding region of nsp1. Screenshots were taken from [32] and modified. (**B**) Schematic representation of nsp1 domain structure [12] and position of mutations K47R and S135R. (**C**) Constructs for dual luciferase reporter assays with co-transfection of eGFP or SARS-CoV-2 nsp1 wt and mutants S135R and K47R/S135R. (**D**) Dual luciferase reporter assay in HEK293 cells with wt or variants of full-length SARS-CoV-2 5′-UTRs and co-transfection of nsp1 (wt and mutants) or eGFP (*n* = 4). Firefly luciferase activity was normalized to *Renilla* luciferase activity (ratio fLuc/hRluc). The ratio of fLuc and hRluc in the presence of nsp1 wt or mutants was normalized to the respective eGFP co-transfection. Statistical significance was calculated using Student’s *t*-test (two-tailed, paired), (**) *P*-value <.01, (*) *P*-value <.05, ns = not significant. (**E**) Representative western blot against nsp1 in HEK293 cells. The relative protein level was calculated by normalizing nsp1 mutants S135R and K47R/S135R to the wt (*n* = 3) (see Supplementary Fig. S4).

With the emergence of BA.2 and following Omicron variants, mutations U670G (Codon: AGU → AGG) and A405G (Codon: AAA → AGA) in the coding region of nsp1 were reported to occur simultaneously with the C21U mutation in SL1 (Fig. 2A and Supplementary Fig. S3). Both mutations lead to proteins with changed amino acids. S135R is located in the highly disordered linker and K47R in the globular NTD (Fig. 2B). Interestingly, both residues are located in predicted interaction sites with SL1 [58]. Mutant S135R is present in all circulating variants to date, while the double mutant K47R/S135R occurs irregularly in different Omicron strains (Supplementary Fig. S3).

We tested the functional relevance of different combinations of SL1 and nsp1 wt and mutants in protein synthesis. For that, we co-expressed the dual luciferase vectors with full-length SARS-CoV-2 5′-UTR variants with either eGFP (control) or with SARS-CoV-2 nsp1 wt, mutant S135R, or double mutant K47R/S135R in HEK293 cells (Fig. 2C). Only minor changes occurred in cells expressing nsp1 wt together with tested SARS-CoV-2 5′-UTR variants. Notably, however, luciferase activity of SARS-CoV-2 5′-UTR variants carrying mutation C21U was significantly reduced in cells co-expressing nsp1 mutants S135R or K47R/S135R (Fig. 2D). This effect for both nsp1 mutants was exclusively found in the presence of mutation C21U in SL1 and not with mutations C241U or C44U/C241U alone. It is caused by a decrease in firefly signal and thus the differential ability of the C21U mutant for mediating escape, and not by differential repression of *Renilla* luciferase as our intrinsic control. Given the role of nsp1 in mRNA degradation, we assessed whether differences in firefly signal are accompanied by concomitant changes at reporter mRNA level. Indeed, C21U mutants showed slightly lower mRNA levels only with nsp1 mutants S135R and K47R/S135R in comparison to wt SL1 (Supplementary Fig. S4A). Notably, nsp1 mutants S135R and K47R/S135R showed similar effects on luciferase activity and mRNA levels, suggesting that the effect is caused by the S135R mutation. Given that the variant UTRs showed comparable translation efficiencies (Supplementary Fig. S2D) and protein levels of the different nsp1 variants were similar (Fig. 2E and Supplementary Fig. S4B), both mutation C21U in SL1 and mutations S135R in nsp1 seem to have a functional influence on the escape from nsp1-mediated repression.

### Pyrimidine composition in the apical loop of SL1 determines RNA dynamics

Since a single nucleotide C to U mutation in the conformationally flexible SL1 apical loop resulted in changed RNA dynamics and function, we further investigated the pyrimidine composition of the 5′-SL1 pyrimidine loop by mutational studies. Previous studies have reported essential features of SL1 and a G-free stretch necessary for escaping nsp1-mediated repression [24, 27, 28]. However, combined data on the structural and functional characteristics of SL1 and the role of the sequence of the pyrimidine loop are missing.

**Figure 3.**
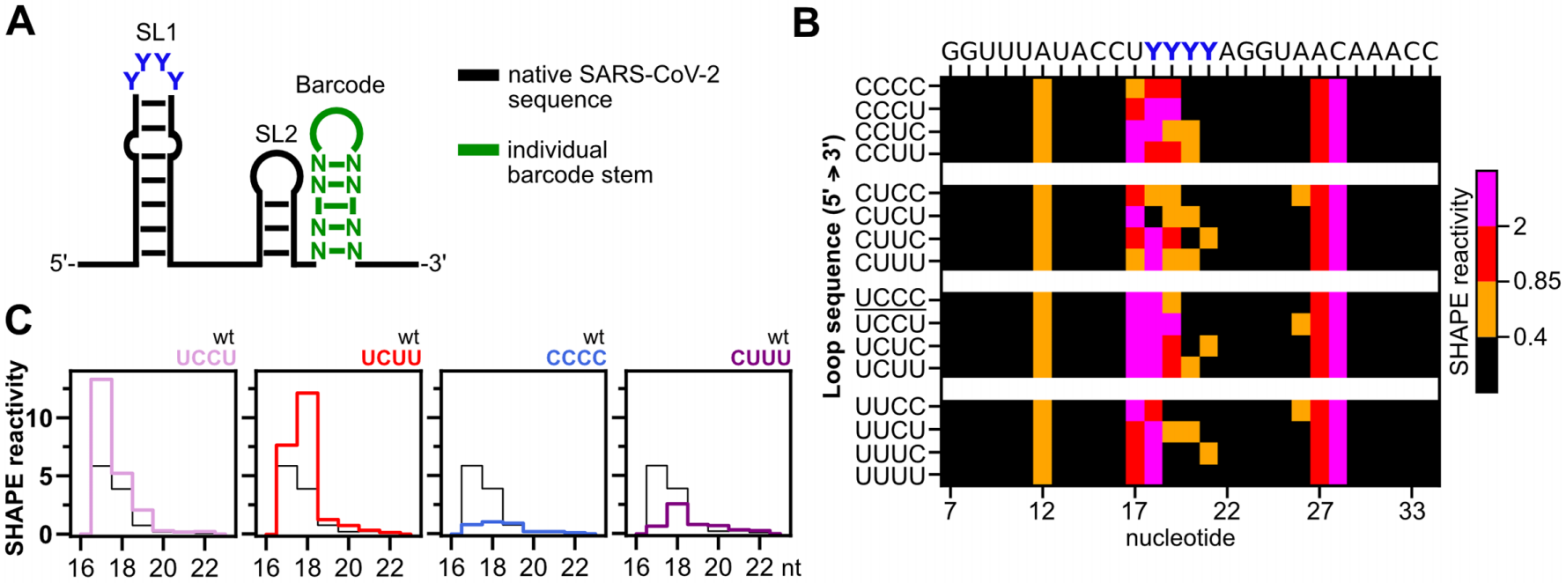
Pyrimidine loop composition of SL1 determines RNA dynamics of the apical loop and the closing base pair. (**A**) Schematic representation of RNA constructs used for SHAPE-MaP containing the native SARS-CoV-2 sequence context and individual barcode stems for each construct (Supplementary Table S2). (**B**) Heat map of SHAPE reactivities of all 16 pyrimidine combinations of the SL1 apical loop. The heat map represents the nucleotides 7–33 of SL1. SL1 wt apical loop sequence UCCC is underlined. (**C**) Comparison of SHAPE reactivities of the wt (black) and respective 5′-SL1 mutants for the nucleotides 16–23 of the apical loop.

To assess the importance of the SL1 loop pyrimidine sequence and structural dynamics for SL1 function, we systematically varied the apical loop pyrimidines to all 16 possible combinations. The 16 pyrimidine combinations were tested for their structural impact by *in vitro* SHAPE-MaP (Fig. 3) and their impact on translation efficiency and escape from nsp1-mediated repression in reporter gene assays. SHAPE-MaP confirmed the overall formation of the SL1 bipartite stem-loop structure for the 16 constructs. Strikingly, each of the 16 constructs exhibited its own unique SHAPE reactivity profile for nucleotides 17–21, emphasizing the dynamic character of the apical loop and enabling an individual investigation of loop dynamics in response to each mutated residue (Fig. 3B, Supplementary Fig. S5, and Supplementary Table S7).

SHAPE reactivity profiles of the wt loop (UCCC) and mutation C21U (UCCU) matched the data from the previous full-length 5′-UTR SHAPE experiments. Across the 16 constructs, in general higher reactivities were observed especially for nucleotides U17, Y18, and, in some cases, for Y19. The individual reactivities at these three positions varied strongly dependent on the pyrimidine composition of the loop. Y20 and Y21 were non- or low-reactive in all constructs.

We identified common patterns emerging from the SHAPE data, suggestive of adjacent nucleotides affecting each other. Constructs with U18 (UYYY) consistently showed high (>0.85) or hyper-reactivity (>2) of nucleotides 17 and 18, while no consistent trend for loop sequences with C18, which displayed diverse reactivity patterns, was observed. This effect suggests a possible adjacent interaction of U18 with U17 in the tested loop constructs. In motifs with U18/C19 (UCYY), nucleotides 17 and 18 were always hyper-reactive, while for U18/U19 motifs (UUYY), only one of the two positions showed hyper-reactivity, while the other one remained highly reactive. Thus, a cytidine at position 2 of the tetraloop enhances the adjacent interaction of U17 and U18.

Further, in constructs combining C19 and U21 (YCYU), all three nucleotides, 17, 18, and 19, were highly (>0.85) or hyper-reactive (>2), with residue 19 only found to be hyper-reactive in the case of YCCU motifs. Nucleotides 20 and 21 were non-reactive in YCCY and YYCC motifs, while residue 21 was exclusively reactive in three of the four possible YYUC motifs. Notably, U17 in UCCU (C21U variant) and U18 in UCUU showed the highest reactivities of all tested constructs with SHAPE reactivities of 12–13 (Fig. 3C). Overall, the motifs CUUU and CCCC showed the lowest SHAPE reactivity for residue 17 across all tested constructs. Furthermore, CCCC and CUCC were the only constructs in which none of the nucleotides was hyper-reactive. Thus, the SHAPE data support the formation of a highly dynamic apical loop in which the pyrimidine composition directs specific SHAPE reactivities for nucleotides 17–21.

To further elucidate the local RNA dynamics of SL1 in response to the pyrimidine composition, we focused on the constructs UCCU (C21U), UCUU, CCCC, and CUUU, as these demonstrated distinct effects in SHAPE data. We performed CD spectroscopy experiments to assess structural changes and thermal stability of the apical loop pyrimidine mutants of SL1 in direct comparison to the wt. Typical A-form double-stranded RNA exhibits a characteristic CD spectrum with a positive maximum around 260–270 nm and a negative minimum near 230–240 nm [59]. Single-stranded RNAs, in contrast, show a broader, less-intense positive band around 260–270 nm and lack a pronounced negative minimum, reflecting a more flexible or unstructured conformation [60]. CD spectra recorded at 10°C, 25°C, and 37°C showed the expected positive maximum between 260 and 270 nm confirming folding of SL1 (Supplementary Fig. S6). The CD signal intensity was highest at 10°C and progressively decreased with increasing temperature, consistent with thermal destabilization or increased structural flexibility at elevated temperatures. SHAPE data confirmed the overall fold of SL1 for all tested constructs. This is in line with the CD spectra showing that both wt and mutant constructs experience similar temperature-dependent changes in their secondary structures.

Additionally, melting temperature analysis (Table 1 and Supplementary Fig. S7) showed that construct CCCC exhibited the highest thermal stability among the tested constructs, with a melting point of 68.1 ± 0.6°C (Supplementary Fig. S7B). The mutants UCCU, UCUU, and CUUU displayed overall reduced melting temperatures compared to the wt (66.5 ± 0.5°C), indicating that the introduced sequence changes marginally destabilize the global RNA structure. Here, UCUU (65.6 ± 0.3°C) and CUUU (65.3 ± 0.2°C) showed melting behaviors closest to the wt, suggesting relatively preserved structural integrity. UCCU (C21U), with a melting point of 64.7 ± 0.3°C, exhibited the most pronounced destabilization among the tested constructs (Supplementary Fig. S7D). This effect is consistent with the formation of a non-canonical U•U interaction in the apical loop, which is energetically less stable than canonical Watson–Crick base pairs and can disrupt optimal base stacking adjacent to the closing base pair, thereby lowering the overall melting temperature [61, 62].

The melting temperature analysis supports the observed SHAPE reactivity profiles of respective pyrimidine constructs. CCCC shows reduced SHAPE reactivity among all constructs arguing for reduced RNA dynamics and flexibility. Consistently, melting temperature analysis showed CCCC to be thermally more stable compared to the wt. UCUU and UCCU showed lower melting temperatures, which indicate a decrease in structural integrity as well as global destabilization. In SHAPE data, these candidates showed the highest SHAPE reactivities for residue 17 (UCCU) or 18 (UCUU), arguing for pronounced RNA dynamics as the origin of decreased thermal stability.

In sum, CD as well as SHAPE structural data imply that the pyrimidine composition does not interfere with general stem-loop formation, but it influences global SL stability as well as the local structure of the apical loop, most likely due to changed structural dynamics and altered base stacking.

### Pyrimidine sequence of the SL1 apical loop influences the escape from nsp1-mediated repression

Next, we were interested in how the pyrimidine composition of SL1 influences the escape from nsp1-mediated repression to understand the structural and functional relevance of SL1 apical loop pyrimidine composition in a cellular context. To investigate the escape independent of nsp1 repression, we used a single firefly luciferase gene instead of the dual luciferase reporter. This single reporter system does not include a second luciferase as intrinsic control, which is repressed by nsp1. Thus, using the single reporter assay allows us an independent investigation and quantification of the escape from nsp1-mediated repression.

**Figure 4.**
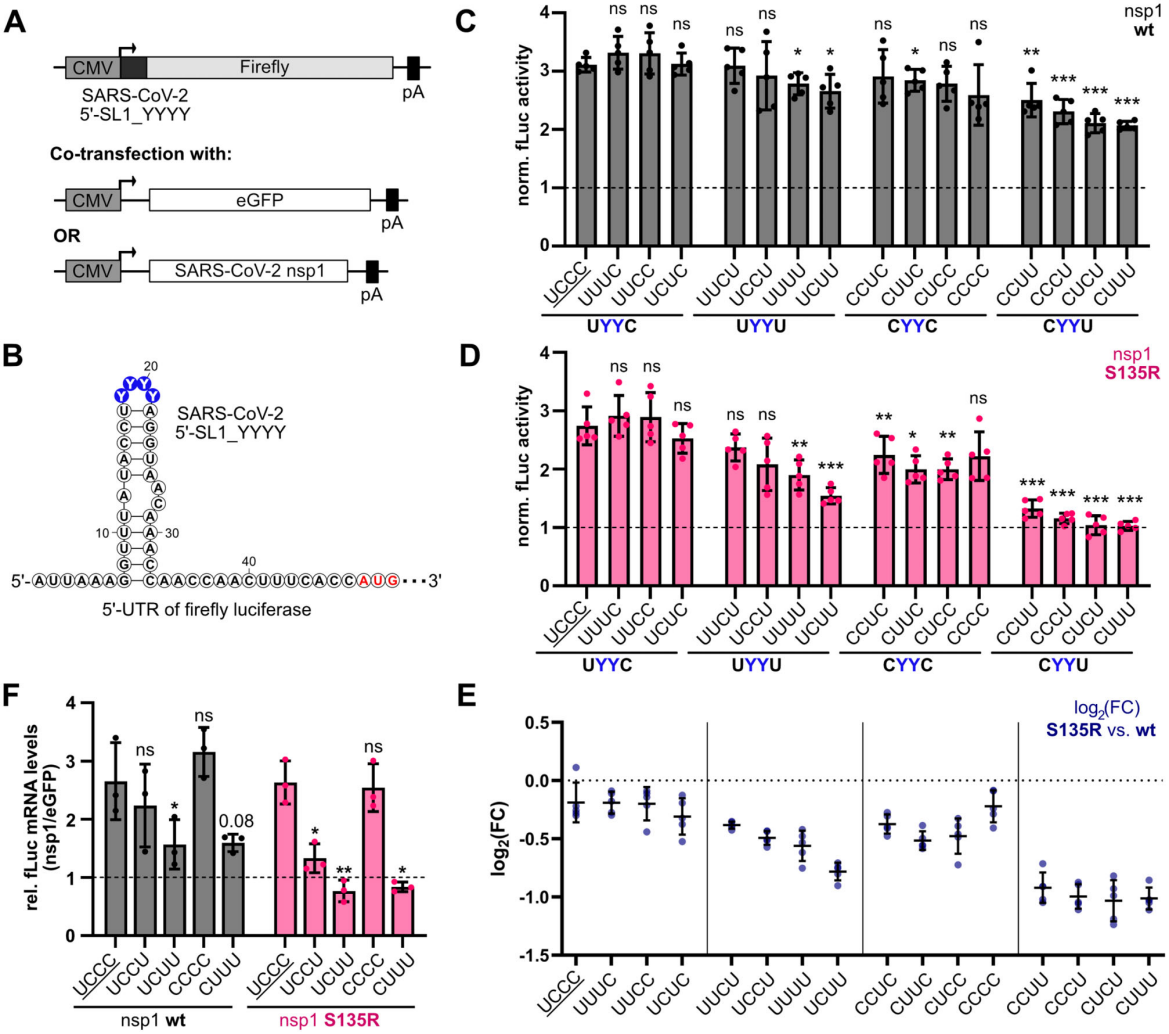
The pyrimidine composition of the SARS-CoV-2 SL1 apical loop influences the escape from nsp1-mediated repression. (**A**) Constructs for single luciferase reporter assays with co-transfection of eGFP or SARS-CoV-2 nsp1 wt and mutant S135R. (**B**) Secondary structure and sequence of the 5′-UTR of the firefly luciferase coding region (fLuc) used for single luciferase reporter assays. Systematically varied pyrimidines of the SARS-CoV-2 SL1 apical loop are colored in blue; the start codon is colored in red. (**C**) Single luciferase assay in HEK293 cells with SL1_YYYY constructs and SARS-CoV-2 nsp1 wt (*n* = 5). SL1 apical loop wt sequence is underlined. Firefly luciferase activity with SARS-CoV-2 nsp1 wt was normalized to the firefly activity of the respective construct with eGFP co-transfection. The dashed line represents the activity in the presence of eGFP. Statistical significance was calculated using Student’s *t*-test (two-tailed, paired), (***) *P*-value <.001, (**) *P*-value <.01, (*) *P*-value <.05, ns = not significant. (**D**) Single luciferase assay in HEK293 cells with SL1_YYYY constructs and SARS-CoV-2 nsp1 S135R (*n* = 5). SL1 apical loop wt sequence is underlined. Firefly activity and statistical significance were calculated as described in panel (C). The dashed line represents the activity in the presence of eGFP. (**E**) Log_2_(FC) of normalized luciferase activity of nsp1 mutant S135R to nsp1 wt. SL1 apical loop wt sequence is underlined. Dashed line represents no fold change between nsp1 wt and mutant S135R. (**F**) Relative fLuc mRNA levels of depicted SL1 single luciferase constructs in HEK293 cells in co-expression with nsp1 wt or mutant S135R (*n* = 3). Statistical significance was calculated as described in panel (C).

The reporter construct contained only SL1 in its 5′-UTR. It was either co-transfected with eGFP (control) or nsp1 wt or mutants S135R and K47R/S135R (Fig. 4A and B, and Supplementary Fig. S8). Normalizing the luciferase activity to eGFP co-transfection (indicated by dashed lines in Fig. 4C and D) reports on the effect of nsp1 co-expression. Values >1 show an enhancement of luciferase activity in the presence of nsp1 and thus escape from repression, while values <1 indicate repression by nsp1.

All pyrimidine loop constructs showed the ability to escape nsp1-mediated repression with enhanced (>1) or comparable (~1) normalized luciferase activities relative to eGFP co-transfected HEK293 cells. However, the efficiency of escape was sensitive to the pyrimidine sequence in the loop of SL1. Interestingly, while nsp1 wt showed only low sensitivity to the tested pyrimidine variants (reduction up to ~30%) (Fig. 4C), nsp1 mutant S135R showed higher sensitivity to the pyrimidine loop composition (reduction up to ~60%) in comparison to the wt (Fig. 4D). The observed reduced enhancement of the nsp1-mediated escape was again not a function of reduced nsp1 protein levels, which were moderately increased for the nsp1 mutant S135R in comparison to the wt (Supplementary Fig. S8C and D). The nsp1 double mutant K47R/S135R behaved identically to the S135R mutant (Supplementary Fig. S8), suggesting that the S135R mutation modulates SL1-mediated escape, while K47R plays only a subordinate role. Thus, in the following, we only discuss the nsp1 single mutant further.

We observed a strong sequence preference for the positioning of uridines and cytidines in the apical loop for all nsp1 variants. The weakest candidate to escape nsp1-mediated repression was the pyrimidine sequence reverse to the wt loop (UCCC → CUUU). When ranking the pyrimidine loop compositions in order of their efficacy to escape nsp1-mediated repression, we found that sequences with U18/C21 (UYYC) showed overall high and unchanged escape compared to the wt (UCCC). In contrast, all nsp1 variants showed the highest sensitivity to candidates with the reverse pattern at positions 1 and 4 in the apical loop, C18/U21 (CYYU). A clear pyrimidine sequence preference for positions 19 and 20 could not be observed. The remaining eight constructs (UYYU and CYYC motifs) showed intermediate escape. Thus, positions 1 (U18) and 4 (C21) of the tetraloop seem to be most important for efficient escape from nsp1 repression.

The sequence clustering was further supported by the comparison of the log_2_-fold changes of the luciferase activities for all constructs in co-expression with nsp1 wt or nsp1 S135R (Fig. 4E). In general, all SL1 mutants showed lower luciferase activity with nsp1 S135R in comparison to nsp1 wt. While UYYC constructs showed an overall lower log_2_-fold change of −0.2 to −0.3, the CYYU cluster was highly and consistently reduced in the presence of nsp1 mutant S135R with a log_2_-fold change of approximately −1.0 in comparison to nsp1 wt. The remaining eight UYYU and CYYC motifs exhibited different abilities within clusters to escape from nsp1 wt or mutants. Here, UYYU motifs showed the most differential response comparing nsp1 S135R to wt (Fig. 4E). The log_2_-fold changes ranged from −0.4 to −0.8 with UCUU showing the highest reduction with S135R. The CYYC motifs displayed overall constant luciferase activity (Fig. 4C and D), and comparison of the nsp1 wt and S135R showed only lower log_2_-fold changes, more similar to the best UYYC motifs. Especially, CCCC approached the wt motif, with only smaller differences in activity between nsp1 wt and S135R.

To exclude that the observed effects were due to overall changes in the translation efficiency of the respective loop mutations, we additionally performed dual luciferase reporter gene assays for all SL1 constructs in the absence of nsp1 (Supplementary Fig. S9A and B). Overall, we did not observe strong effects on translation efficiency of different SL1 constructs in comparison to the wt SL1. Especially constructs with reduced nsp1 S135R vs. nsp1 wt activity did not show significant differences in translation efficiency in comparison to the wt.

Further, we assessed whether the observed differences in luciferase activity are accompanied by changes in mRNA levels. Thus, we quantified mRNA levels for the naturally occurring loop variants UCCC (wt) and UCCU (C21U), the CUUU mutant, which showed the lowest escape, and the UCUU and CCCC mutants that deviate most from their respective motif cluster; these were co-transfected with eGFP, nsp1 wt or nsp1 S135R. In accordance with the observed changes of firefly signal, we found increased mRNA levels for loop variants mediating efficient escape (UCCC, UCCU, and CCCC) and lower mRNA levels for the less effective loop variants UCUU and CUUU (Fig. 4F). Similar to the effect on firefly signal, the reduction at mRNA levels is more pronounced for the nsp1 S135R mutant than for the nsp1 wt, suggesting that the mutation S135R does not affect overall nsp1 function, but specifically modulates the escape from nsp1-mediated repression.

Taken together, cellular data showed a functional relevance of the order of the pyrimidines in the apical loop for the escape from nsp1-mediated repression. Underlying the observed preference for specific pyrimidine combinations at positions 1 and 4 of the apical loop, a structural relevance of SL1 is apparent when focusing on the candidates that deviate within their respective motif clusters, especially UCUU and CCCC. These two candidates showed noticeable SHAPE reactivity characteristics, with UCUU having the highest SHAPE reactivity at position 18 among all constructs and CCCC showing overall reduced reactivity in comparison to the wt UCCC. Accordingly, melting temperature analysis of the CCCC SL1 variant showed a higher thermal stability compared to the wt. Thus, cellular data suggest that nsp1 mutants S135R and K47R/S135R are more sensitive to loop dynamics than the nsp1 wt. While the more stable CCCC variant is well able to confer escape from repression also for the nsp1 mutants, the highly reactive UCUU loop is less able to mediate escape.

### Structure and sequence of SL1 are both essential for escaping nsp1 activity

**Figure 5.**
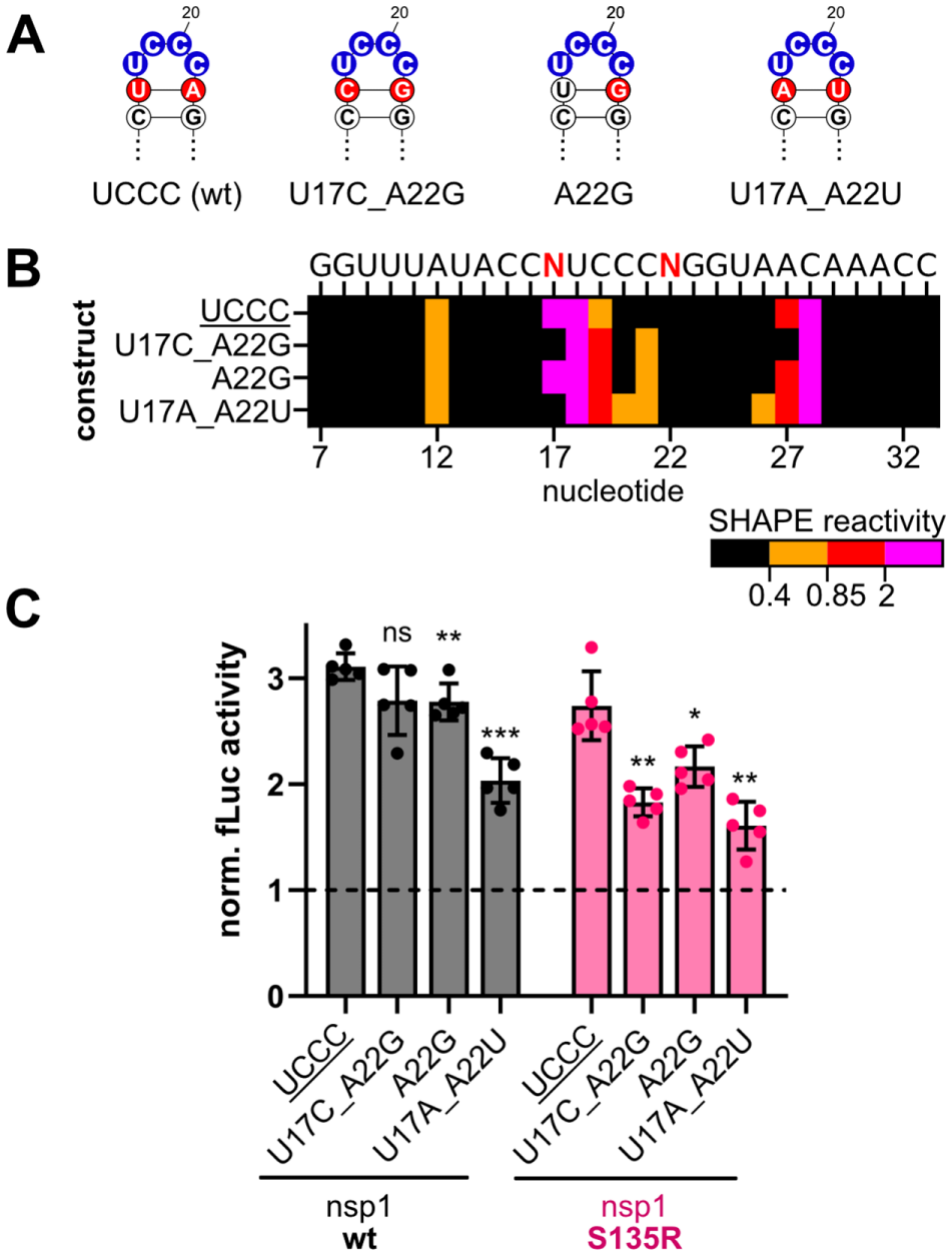
The closing base pair influences the structural dynamics of the SARS-CoV-2 SL1 and the escape from nsp1-repression. (**A**) Tested mutations of the closing base pair of SARS-CoV-2 SL1 in SHAPE-MaP and luciferase experiments. (**B**) Heat map of SHAPE reactivities of the closing base pair mutants of SARS-CoV-2 SL1. The heat map represents the nts 7–33 of SL1. SL1 apical loop wt sequence is underlined. (**C**) Single luciferase assay in HEK293 cells with SARS-CoV-2 SL1 closing base pair constructs and SARS-CoV-2 nsp1 wt and mutant S135R (*n* = 5). The same single reporter gene assay was used as shown in Fig. 4A. Firefly activity was calculated as described in Fig. 4C. SL1 apical loop wt sequence is underlined. The dashed line represents the activity in the presence of eGFP. Statistical significance was calculated using Student’s *t*-test (two-tailed, paired), (***) *P*-value <.001, (**) *P*-value <.01, (*) *P*-value <.05, ns = not significant.

To gain deeper insight into the importance of the structural flexibility of the apical loop of SL1 to mediate escape from nsp1, we performed a more detailed structure–function analysis. Since we observed strong SHAPE reactivity changes at position U17, we first tested the structural and functional contribution of the labile base pair of the upper helix of SL1. We therefore mutated nts 17 and 22 to alter base pairing (Fig. 5A). Here, we avoided the introduction of guanosines in the presumably essential G-free sequence window to allow for unbiased cellular testing [27]. Introducing a C-G base pair as a closing base pair (U17C/A22G) resulted in a non-reactive cytidine at position 17 in contrast to the highly reactive uridine in the wt (Fig. 5B, Supplementary Fig. S5, and Supplementary Table S7). This is in line with a stabilization of the base pair and altered structural dynamics of the loop in response to the introduced C-G base pair. Closing this upper base-pair seems to translate into stabilization of the complete SL1, as SHAPE also shows loss of reactivity in the internal loop. In contrast, destabilization of the closing base pair by introducing a G at position 22 (A22G), resulting in a U۰G wobble, led to a comparable SHAPE reactivity profile as the wt (Fig. 5B). This underlines the labile and partially open character of the U-A base pair according to the SHAPE reactivity data. Switching of the U-A to an A-U base pair (U17A/A22U) induced a remarkable loss of reactivity for position 17 (Fig. 5B). This likely corresponds to a changed loop conformation as well as stacking interaction of the switched bases. The reduction of reactivity at nucleotide 17 in the mutant U17A/A22U is in line with the previous observation of highly or hyper-reactive nucleotides at nucleotides 17 and 18 in U18 motifs (UYYY), suggestive of an interaction between U18 and U17. Strikingly, U17A/A22U was the only construct in which all of the loop nucleotides became reactive. This underlines the interconnection of the closing base pair and the pyrimidine loop for the structural dynamics of the SL1 apical loop.

Testing these constructs in reporter assays (Fig. 5C), we observed the strongest effect on escape from nsp1-mediated repression for construct U17A/A22U for both nsp1 wt and mutant S135R, having decreased luciferase activity in comparison to the SL1 wt. This might signify the importance of an uninterrupted pyrimidine stretch for escape from SARS-CoV-2 nsp1 activity. Interestingly, nsp1 S135R was more sensitive to both stabilization and destabilization of the closing base pair, arguing for a stringent sensitivity of mutant S135R to structural dynamics of the apical loop. Again, these effects were consistent for nsp1 mutant K47R/S135R (Supplementary Fig. S8E). Additionally, these differences did not result from altered translation efficiencies of the SL1 closing base pair constructs, as all three constructs show overall unchanged translation efficiency in comparison to the SL1 wt in dual luciferase assays (Supplementary Fig. S9B).

**Figure 6.**
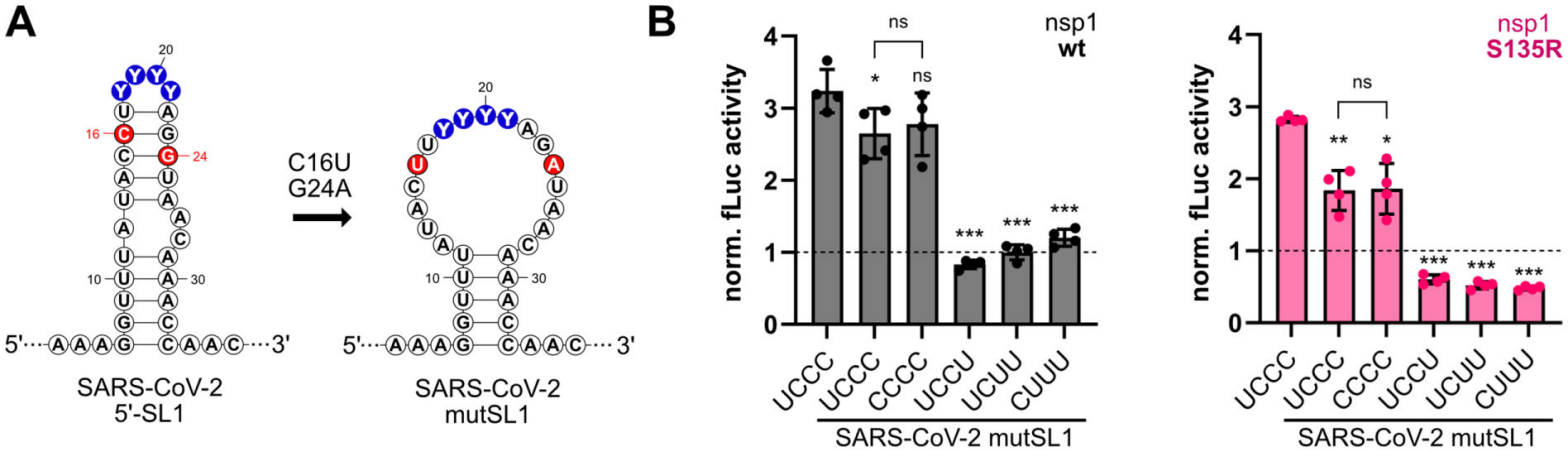
Both structure and sequence are essential for the escape of SARS-CoV-2 nsp1 activity. (**A**) Secondary structure of SARS-CoV-2 SL1 and mutated SARS-CoV-2 SL1 (SARS-CoV-2 mutSL1) with mutations C16U and G24A (colored in red) used in single luciferase reporter experiments. (**B**) Single luciferase assay in HEK293 cells with SARS-CoV-2 mutSL1 constructs and SARS-CoV-2 nsp1 wt and mutant S135R (*n* = 4). The same single luciferase assay was used as described in Fig. 4A. SL1 apical loop wt sequence is underlined. Firefly activity was calculated as described in Fig. 4C. The dashed line represents the activity in the presence of eGFP. Statistical significance was calculated using Student’s *t*-test (two-tailed, paired), (***) *P*-value <.001, (**) *P*-value <.01, (*) *P*-value <.05, ns = not significant.

In the next step, we compared how the structurally and functionally noticeable SL1 loop motifs UCCC (wt), UCCU (C21U), CCCC, UCUU, and CUUU behave in a destabilized, open SL context. We introduced two minimal mutations, C16U and G24A, to disrupt upper stem formation and to create five mutSL1 constructs, respectively (Fig. 6A). The mutations were chosen to keep the pyrimidine stretch of SL1 and the lower stem region intact.

Testing the mutSL1 constructs in single luciferase assays (Fig. 4A) with nsp1 wt and mutant S135R, we found distinct effects on evasion from nsp1-mediated repression in the absence of the upper stem (Fig. 6B). Motifs UCCC and CCCC in mutSL1 still showed escape both with nsp1 wt and nsp1 S135R. Notably, mutant S135R showed significantly lower enhancement of UCCC and CCCC in mutSL1, while nsp1 wt showed comparable enhancement compared to intact SL1 wt, again demonstrating higher sensitivity of the S135R mutant and robustness of nsp1 wt against structural changes. In contrast, motifs UCCU, UCUU, and CUUU showed a strong decrease in luciferase activity in the mutSL1 context, compared to their respective intact SL1 sequence counterparts (compare Fig. 4C and D, and Fig. 6B). Again, these differences were not the result of an overall different translation efficiency of the SARS-CoV-2 mutSL1 constructs (Supplementary Fig. S9C).

Thus, the results demonstrate that specific pyrimidine sequence motifs can be sufficient to mediate the escape from nsp1 repression, while a random pyrimidine sequence alone is not able to mediate efficient escape. At the same time, the data also indicate that non-ideal pyrimidine sequences can still escape nsp1-mediated repression in the SL1 structural context, while they cannot if the SL structure is missing, most evidently for UCCU (C21U). In consequence, together preferred sequence and structural features of SL1 mediate escape from nsp1 repression.

### Co-evolution of SL1 and nsp1 in SARS-CoV and SARS-CoV-2

**Figure 7.**
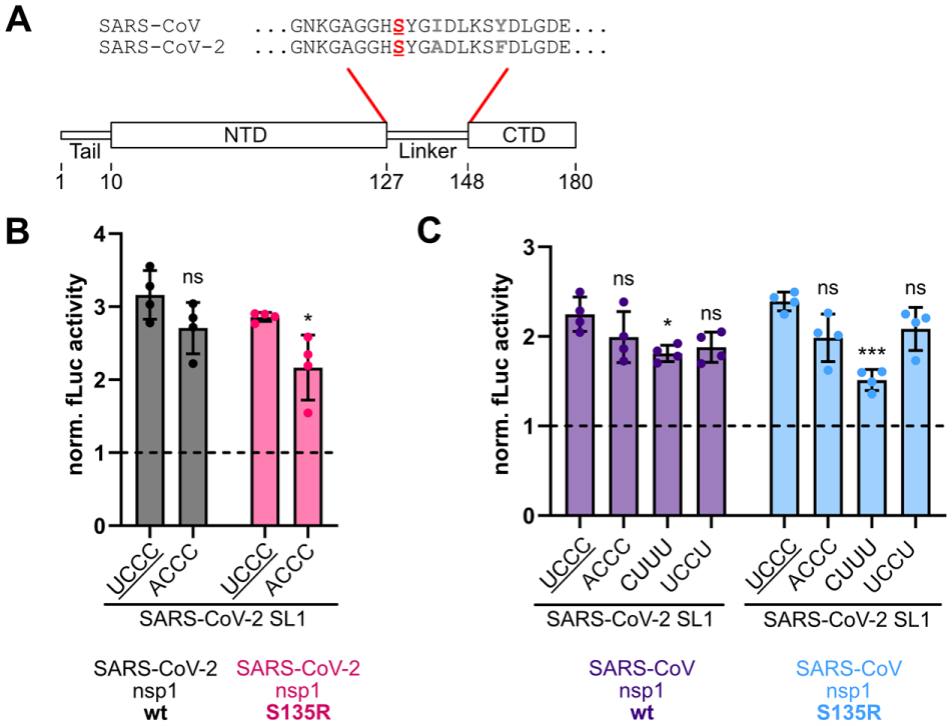
SARS-CoV nsp1 mutant S135R shows similar sensitivity to SARS-CoV-2 SL1 apical loop composition. (**A**) Schematic representation of nsp1 domain structure [12] and alignment of the amino acid sequence of the linker region of SARS-CoV and SARS-CoV-2 nsp1 using Clustal Omega [64]. Serine at position 135 is highlighted in red. Differing amino acids between SARS-CoV and SARS-CoV-2 nsp1 are colored in gray. (**B**) Single luciferase assay in HEK293 cells with SARS-CoV-2 SL1 wt and mutant U18A (ACCC) and SARS-CoV-2 nsp1 wt and mutant S135R (*n* = 4). SARS-CoV-2 SL1 apical loop wt sequence is underlined. Firefly activity was calculated as described in Fig. 4C. The dashed line represents the activity in the presence of eGFP. Statistical significance was calculated using Student’s *t*-test (two-tailed, paired), (*) *P*-value <.05, ns = not significant. (**C**) Single luciferase assay in HEK293 cells with SARS-CoV-2 SL1 constructs and SARS-CoV nsp1 wt and mutant S135R (*n* = 4). The *x*-axis shows the nucleotide compositions of the SARS-CoV-2 SL1 apical loop. SARS-CoV-2 SL1 apical loop wt sequence is underlined. Firefly activity and statistical significance were calculated as described in panel (B). The dashed line represents the activity in the presence of eGFP. Statistical significance was calculated using Student’s *t-*test (two-tailed, paired), (***) *P*-value <.001, (*) *P*-value <.05, ns = not significant.

It has been shown that SL1 and nsp1 from different coronaviruses co-evolve to mediate efficient escape for their own genome and sub-genomic mRNAs [31, 63]. Thus, to understand the ongoing co-evolution of SL1 and nsp1, we compared this interplay to the close SARS-CoV-2 relative SARS-CoV (Fig. 7). SL1 in SARS-CoV carries an adenosine at position 18, changing the apical loop sequence to ACCC, and interrupting the pyrimidine stretch important for the escape of SARS-CoV-2 SL1. Further, the SARS-CoV SL1 differs from SARS-CoV-2 SL1 by a shorter upper stem and a single nucleotide bulge (A14) instead of an asymmetrical internal loop. Our findings suggested that the U at position 1 of the apical loop contributes to escape from nsp1, especially for the more sensitive S135R variant. Indeed, introducing an A at position 18 (ACCC) resulted in a reduction of luciferase activity for SARS-CoV-2 nsp1 S135R compared to SL1 wt (Fig. 7B). SARS-CoV-2 nsp1 wt showed no significant change in escape, again demonstrating the higher sensitivity of the nsp1 mutant toward smaller sequence and structural changes. Interestingly, the *vice versa* experiment exchanging the ACCC to a UCCC in the apical loop of the SARS-CoV SL1 and thus restoring the pyrimidine stretch did not result in enhanced escape of SARS-CoV SL1 (Supplementary Fig. S10). This finding is consistent with our results that the SL context governs the efficiency of a pyrimidine stretch to mediate escape from nsp1. It also suggests that indeed SL1 and nsp1 co-evolve and SARS-CoV nsp1 is sensitive to different RNA features.

Nsp1 from SARS-CoV shares ~84% amino acid sequence identity with nsp1 from SARS-CoV-2 (Fig. 7A and Supplementary Fig. S10A) [12, 64], with the linker position S135 conserved. To gain an initial understanding of the extent to which SARS-CoV nsp1 shares preferences for SL1 with SARS-CoV-2 nsp1, we tested different SARS-CoV-2 SL1 constructs for escape. All SARS-CoV-2 SL1 constructs were able to mediate escape from the SARS-CoV nsp1 wt and an S135R mutant (Fig. 7C). Interestingly, pyrimidine loop variant CUUU, which showed the lowest escape with SARS-CoV-2 nsp1 variants, also showed the lowest escape from SARS-CoV nsp1 repression. In accordance with the data for SARS-CoV-2, this effect was more pronounced for the SARS-CoV nsp1 mutant S135R. Moreover, consistent with SARS-CoV-2 nsp1, the length of the pyrimidine stretch in SARS-CoV SL1 did not affect escape from SARS-CoV nsp1 and the S135R mutant (Supplementary Fig. S10).

Together, these findings suggest that while there is an overlap in the preference for specific pyrimidine motifs between SARS-CoV and SARS-CoV-2 nsp1, these preferences are modulated by the structural context in which they are presented. The differing stem structures between SARS-CoV and SARS-CoV-2 SL1 support this finding since the tetraloop sequences ACCC and UCCC in the structural context of SARS-CoV SL1 do not lead to decreased escape from nsp1-mediated repression for all nsp1 mutants. Further, our data underline the functional relevance of S135R for escape of nsp1-mediated repression, which is conserved between SARS-CoV and SARS-CoV-2.

## Discussion

Our data demonstrate that the pyrimidine sequence specifically directs SHAPE reactivities for nts 17–21 of the apical part of SARS-CoV-2 SL1 and thus determines the RNA dynamics of the pyrimidine loop and the closing base pair. Here, the identified sequence patterns suggest that adjacent nucleotides affect each other. Further, we show apical loop nucleotide positions 1 and 4 and the structural SL1 context to be important for the escape from nsp1-mediated repression.

Our findings underline a clear sequence preference for U at position 1 and C at position 4 within the apical loop of SL1 for the escape from nsp1-mediated activity, which is also dependent on its presentation in the structural SL1 context. Interestingly, SARS-CoV-2 nsp1 wt exhibits a greater tolerance towards sequence and structural variations of the apical loop of SL1, while linker mutant S135R is sensitive even to minor deviations. However, certain sequence motifs (UCCC and CCCC) were able to escape nsp1-mediated repression even when the upper stem of SL1 is disrupted. It has been previously reported that the apical part of SL1 is essential for the escape from nsp1-mediated repression [23], yet our data show that apical stem formation is not strictly necessary when a specific pyrimidine sequence is present and the G-free 5′-proximal sequence window is kept [27]. This might also be the reason why our tested pyrimidine constructs did not indicate a clear preference for cytidines being present at positions 19 and 20, as reported previously with mutants C19G and C20G [24]. Our data strongly suggest that both sequence and structure contexts are necessary for efficient escape. However, specific pyrimidine sequences can also mediate escape from nsp1-repression activity. This is in line with the reported escape of non-5′-SL1-containing mRNAs, such as synthetically derived 5′-UTRs of mRNAs [28] as well as terminal oligopyrimidine (TOP)-motif-containing mRNAs [65]. TOP motifs are single-stranded pyrimidine stretches directly downstream of the 5′-cap with a 5′-cytidine and 4–15 following pyrimidines [66]. Thus, TOP motifs may resemble the pyrimidine stretch in SL1, which leads to escape or at least to a lower repression by nsp1. This is also in accordance with a recent study on the escape of TIAR transcripts from nsp1-mediated repression, confirming a pyrimidine sequence as well as a guanosine-free sequence window to be essential for escape [67].

Circulating SARS-CoV-2 SL1 variants (UCCC or UCCU) comprise a tetraloop consisting only of pyrimidines (4Y), both with a U-A closing base pair. Here, we observed distinct changes in the SHAPE reactivities for nts 17–21, including U17 of the closing base pair, comparing the wt and the mutant loops, arguing for local conformational changes of the loop nucleotides. Further, our SHAPE-MaP results of SARS-CoV-2 SL1 wt demonstrate the flexible and disordered nature of the tetraloop, supported by previous studies [25, 26]. However, we provide further comprehensive structural data on the influence of all 16 possible pyrimidine combinations, presenting deeper insights into the dynamic nature of 4Y loops.

Most common tetraloop folds include canonical GNRA and UNCG as well as GGUG, RNYA, AGNN, and CUUG folds [68, 69]. Pyrimidine pure loops (4Y) as well as U-A closing base pairs are rare among studied naturally occurring RNA tetraloop folds [26, 68, 70]. Our data underline that the loop pyrimidine sequence directs specific SHAPE reactivity patterns for nucleotides 17–21 and thus determines the RNA dynamics in SARS-CoV-2 SL1. Additionally, CD analysis showed that different pyrimidine compositions of the SL1 apical loop do not alter global secondary structure formation of the SL but change thermal stability, most likely influenced by changed loop topologies. Our mutational studies on the closing base pair of SL1 revealed differential SHAPE reactivities for the loop nts 18–21 depending on the orientation of the base pair (U-A and A-U) and the nucleotide identity (C-G). Strikingly, switching the closing base pair from U-A to A-U showed distinct changes in the conformational flexibility of the apical loop as indicated by SHAPE-MaP. Thus, our findings support previous studies on the influence of the closing base pair on the conformational stability and dynamics of RNA tetraloops [70, 71]. In general, RNA tetraloops were reported to be most stable with a C-G closing base pair [71]. The stability of tetraloops with other closing base pairs, like A-U, was reported to depend on the loop sequence [71]. This is also evident for the naturally occurring SL in the histone pre-mRNA 3′-UTR with an apical UUUC tetraloop containing a closing U-A base pair [72, 73]. This 4Y SL adopts a classical A-form helix [72] and, remarkably, the tetraloop was shown to be well organized and stabilized by base stacking interactions of the loop uridines and the closing U-A base pair [72–74]. This is in line with our SHAPE-MaP data of the UUUC tetraloop, which showed only one hyper-reactive nucleotide (position 1 of the loop) but otherwise only lower SHAPE reactivities. However, the high-resolution NMR structure of SL1 shows a high degree of disorder in the UCCC tetraloop [25, 26], indicating that certain sequence variations within the 4Y SL family prohibit a unified structural classification. Due to our comprehensive coverage of all possible 4Y variants, our SHAPE data provide not only insights into the loop dynamics of SARS-CoV-2 SL1 but also into general structural dynamics of 4Y tetraloops toward a better understanding of this rare class of tetraloop folds.

Importantly, our findings demonstrate that the tetraloop mutation C21U of SARS-CoV-2 SL1 not only proves to be structurally relevant, but also shows functional influence on the escape from nsp1-mediated repression in the evolved SARS-CoV-2 nsp1 mutant S135R. The naturally co-evolved double mutant K47R/S135R showed similar behavior in response to SL1 mutations. Thus, while both nsp1 sites 47 and 135 are present in *in silico* predicted interaction regions with SL1 [58], position 135 seems to be more relevant for escape. This is in line with previous findings for SARS-CoV nsp1, where position K47 was shown to be less relevant for the co-immunoprecipitation of SL1-containing transcripts and escape from nsp1-mediated repression [22]. In addition, the exchange of a positively charged lysine for a positively charged arginine at position 47 might preserve the function of this position. However, our data show a clear relevance for the mutation at position 135 that changes a polar and uncharged serine to a positively charged arginine for the escape of nsp1-mediated repression. Position 135 is located in the highly disordered linker region. We show that this naturally occurring nsp1 S135R variant exhibits a clearly different behavior toward the co-occurring SL1 mutant C21U as well as to the SL1 loop pyrimidine composition and SL1 structural features. Thus, we provide first experimental evidence for the *in silico* predicted relevance of position 135 for the presumed SL1/nsp1 interaction. Furthermore, our observations are in line with previous findings that the nsp1 linker region is, in general, important for nsp1 activity and function. The linker region was reported to be relevant for the repression of non-5′-SL1-containing transcripts [29, 30] as well as to be functionally involved in translation inhibition [75]. Previous studies on a SARS-CoV-2 nsp1 variant lacking amino acids K141, S142, and F143 in the linker region underlined the relevance of the linker region for the escape of SL1-containing reporter constructs [30]. In addition, H134 was shown to be functionally relevant for RNA cleavage, further highlighting the importance of the nsp1 linker region for protein function [21].

Therefore, our data suggest that the nsp1 linker governs the preferred translation of viral RNAs and thus might modulate the presumed nsp1/SL1 interaction. Overall, interdomain linkers of RNA-binding proteins were described to be essential for substrate and target affinity e.g. supporting the arrangement of RNA-binding domains [76, 77]. The results of this study therefore support a stronger focus on the contribution of the linker region to nsp1 function, also regarding constantly evolving variant mutations of the protein and evolutionary conservation of closely related coronaviruses.

SL1 and nsp1 sequence conservation were shown to be low between different beta-coronaviruses, although general nsp1 ribosome binding as well as the importance of the 5′-leader for the escape from nsp1-mediated repression were shown to be conserved [31, 75]. For example, MERS-CoV 5′-leader and the HCoV-OC43 5′-leader were shown to fail escaping from SARS-CoV-2 nsp1-mediated repression, arguing for a specific RNA-protein co-evolution [31], supported by studies on the correlation of sequence signatures between the viral 5′-UTR and nsp1 in beta-coronaviruses. Here, mutations introduced in the SARS-CoV-2 nsp1 NTD sequentially resulting in the SARS-CoV nsp1 NTD showed distinct changes in the escape from nsp1-mediated repression [63]. We provide further evidence for this co-evolution of nsp1 and SL1, showing the feature of increased sensitivity of linker mutant S135R in SARS-CoV, again arguing for deeper functional and evolutionary consideration of the nsp1 linker region.

Interestingly, the nsp1 S135R mutant is present in all circulating SARS-CoV-2 variants since its emergence (Fig. 2A). This prevalence suggests that it might provide an advantage in viral fitness. Based on our data that the nsp1 S135R mutant is more sensitive toward sequence and structural changes in SL1, this linker mutation could provide an enhanced discrimination between viral and host mRNAs and thus favor viral replication in cells. Thus, host mRNAs which share sequence features with SL1, but are less or completely unstructured, are more repressed as demonstrated for unstructured SL1 mutants (Fig. 5B). Such a heightened discrimination between self and non-self might be especially relevant in the early steps of infection, where only few viral transcripts are present in the cell. If the C21U mutation in SL1 provides a benefit is less clear. Our measurements show a generally lower protein synthesis in the presence of nsp1 for this SL1 variant. However, it might impact viral functions that we did not monitor, such as viral replication or transcription of subgenomic mRNAs. The C21U mutation initially disappeared after its first emergence and shows larger fluctuations in its occurrence in circulating variants (Fig. 2A). Thus, it is possible that this mutation is under less evolutionary pressure for viral fitness and might reappear due to the generally frequent occurrence of C to U mutations observed for SARS-CoV-2 [78–80].

Importantly, SARS-CoV-2 SL1 and nsp1 were discussed to be promising targets for antiviral therapy by specifically targeting functional SL elements with small molecules [81] or antisense oligonucleotides [24, 30] as well as small molecules disrupting nsp1-ribosome interaction [82]. Our data underline that it is important to assess structural and functional molecular insights into the co-evolutionary process to inform future targeting strategies.

## Supplementary Material

gkag364_Supplemental_Files

## Data Availability

The data underlying this article are available in the article and in its online supplementary material.
