# Supplementary material for: Structure and sequence characteristics of 5′-stem-loop 1 modulate the escape from nsp1-mediated repression in SARS-CoV-2 variants
Source: Nucleic Acids Res. 2026 Apr 28;54(8):gkag364. doi: 10.1093/nar/gkag364 (PMC13122180; doi:10.1093/nar/gkag364)
Supplement: gkag364_Supplemental_Files [file gkag364_supplemental_files.zip › Schoth_Supplementary_Data_revised.pdf]

Supplementary Data

Structure and sequence characteristics of 5'-stem-loop 1 modulate the escape from nsp1-mediated repression in SARS-CoV-2 variants

Julian B. Schoth[1], Inge Schwedt[1], Susanne Philipp[1], Sabrina Toews[2], Leonie Schuebel[1], Anna Wacker[2], Harald Schwalbe[2], Julia E. Weigand[1,3*]

[1] Department of Pharmacy, Institute of Pharmaceutical Chemistry, Marburg University, 35037 Marburg, Germany

[2] Institute for Organic Chemistry and Chemical Biology, Center for Biomolecular Magnetic Resonance (BMRZ), Goethe University Frankfurt am Main, 60438 Frankfurt/Main, Germany

[3] Center for Synthetic Mircobiology (SYNMIKRO), Marburg University, 35043 Marburg, Germany

*To whom correspondence should be addressed. Email: julia.weigand@uni-marburg.de

Contents

Supplementary Figures

Figure S1: SHAPE-MaP data of full-length 5'-UTR of SARS-CoV-2 variants and a coronavirus.......... **3**

Figure S2. SHAPE-MaP data and translation efficiency of the 5'-UTR of SARS-CoV-2 variants.......... **4**

Figure S3: Frequencies of SARS-CoV-2 variants and selected mutations **5**

Figure S4. Relative mRNA levels of full-length SARS-CoV-2 5'-UTR reporters and relative nsp1 protein levels.......... **6**

Figure S5: Full-length heatmap of SHAPE-MaP data of SL1_YYYY constructs and closing base pair mutants.......... **7**

Figure S6: Circular dichroism (CD) spectra of SL1 wt and the depicted SL1 mutants at different temperatures.......... **8**

Figure S7: CD melting curves were recorded at the wavelength corresponding to the highest detected CD signal at 25 °C.......... **9**

Figure S8: Activity of SARS-CoV-2 nsp1 double mutant K47R/S135R in response to the pyrimidine composition of the SARS-CoV-2 5'-SL1 apical loop.......... **10**

Figure S9: Relative translation efficiency in dual luciferase reporter assays of SL1 constructs.......... **11**

Figure S10: Activity of SARS-CoV-2 and SARS-CoV nsp1 wt and mutants with SARS-CoV 5'-SL1.......... **12**

Supplementary Tables

Table S1: Full-length RNA sequences of coronaviral 5'-UTRs for structural probing using SHAPE-MaP. **13**

Table S2: Overviews of barcode sequences and RNA sequences of each SL1_YYYY variant for structural probing using SHAPE-MaP.......... **14**

Table S3: DNA sequences of full-length SARS-CoV-2 5'-UTRs in the dual luciferase reporter gene (hRluc/fLuc) plasmids with bidirectional CMV promoter.......... **16**

Table S4: DNA sequences of 5'-SL1 constructs in the single luciferase reporter gene (fLuc) and dual luciferase reporter gene (hRluc/fLuc) plasmids.......... **17**

Table S5: RNA sequences of tested SL1_wt and C21U (nt 7-33 of SARS-CoV-2 5'-SL) constructs in NMR experiments.......... **19**

Table S6: SHAPE-MaP data of full-length 5'-UTR of SARS-CoV-2 variants.......... **external file**

Table S7: SHAPE-MaP data of SARS-CoV-2 SL1 constructs.......... **external file**

Supplementary References.......... **19**

Supplementary Figures

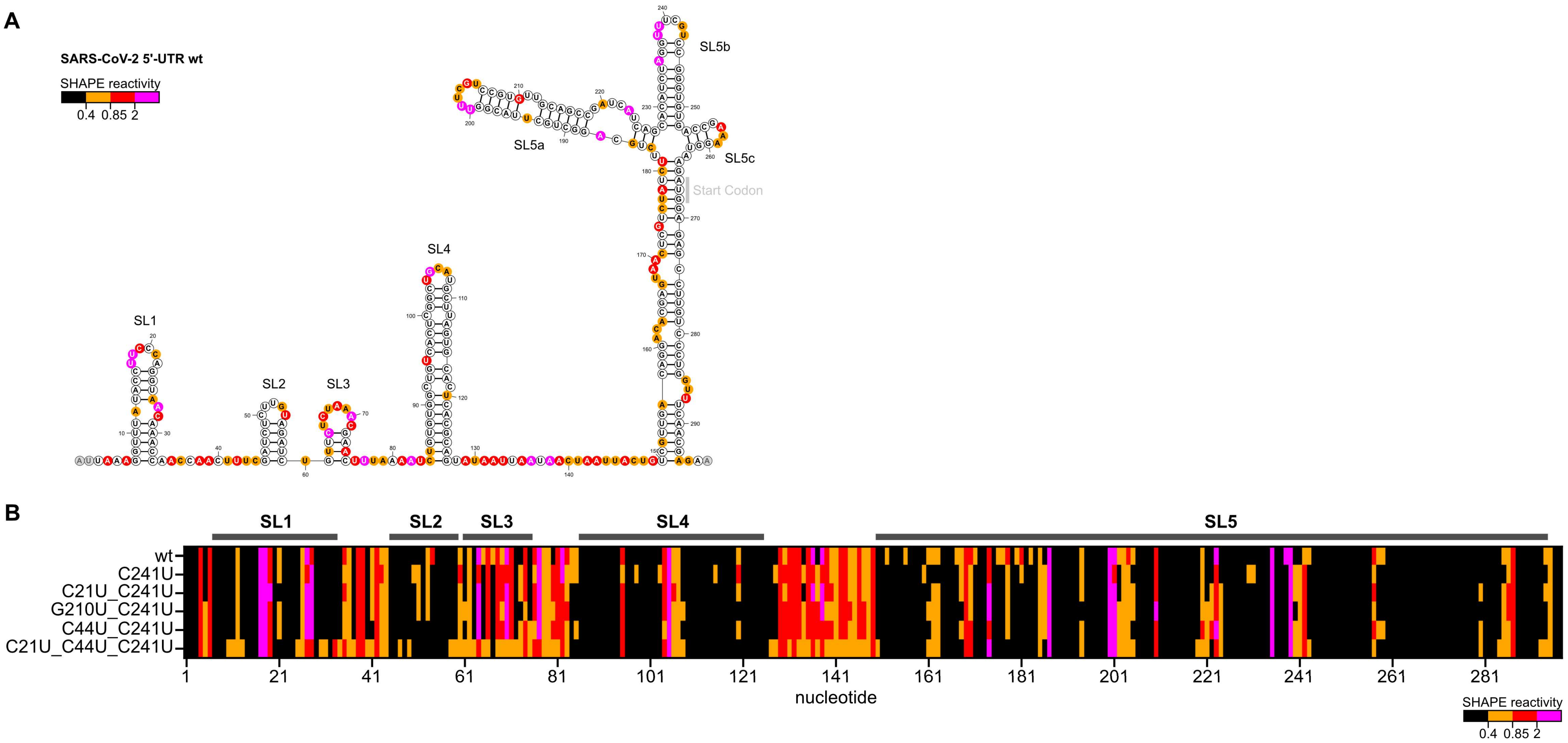

Figure S1. SHAPE-MaP data of the full-length 5'-UTR of SARS-CoV-2 variants. (A) Secondary structure fold prediction of the SARS-CoV-2 5'-UTR wt including SHAPE reactivities. **(B)** Heatmap of SHAPE reactivities of all tested full-length 5'-UTR variants.

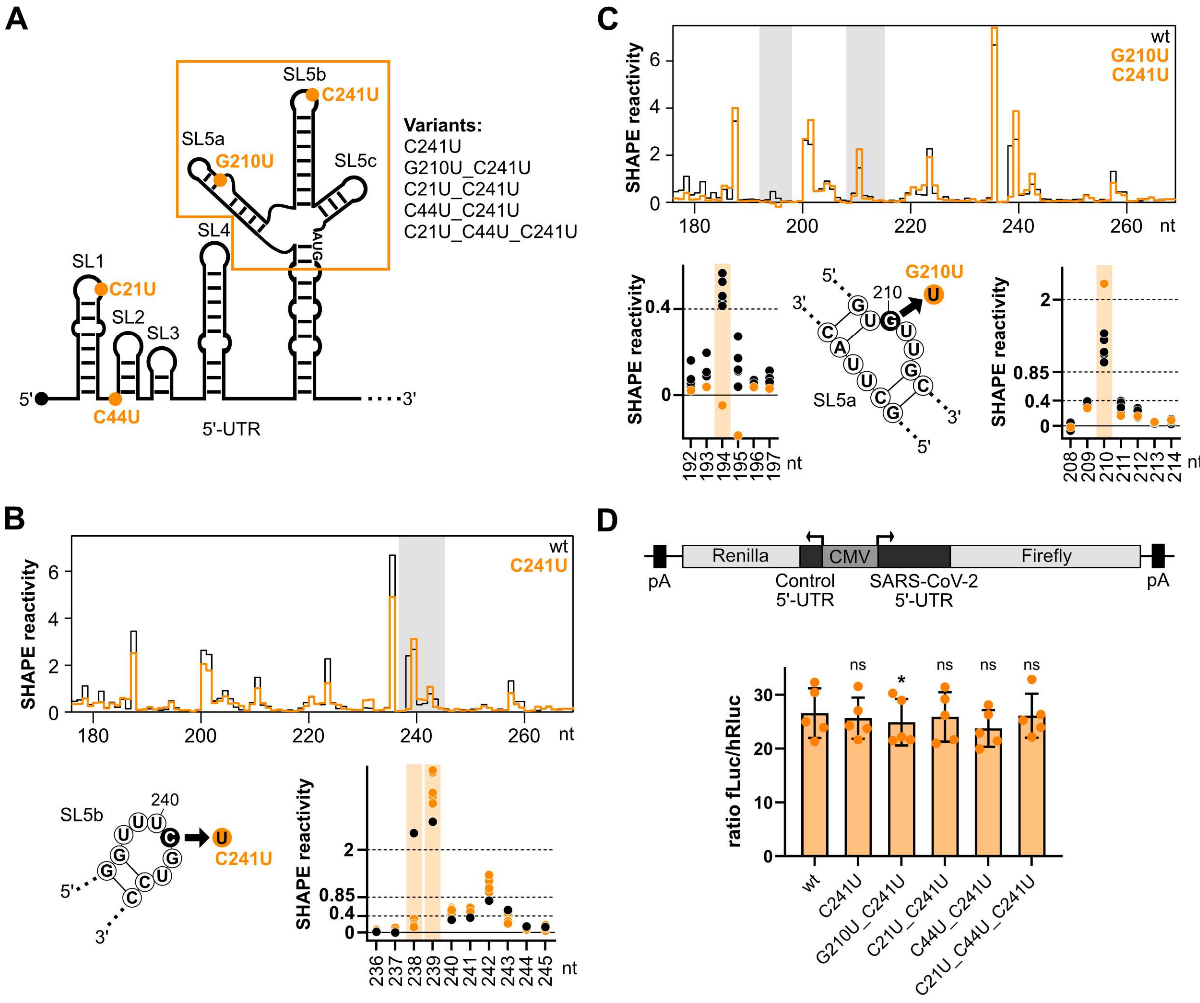

Figure S2. SHAPE-MaP data and translation efficiency of the 5'-UTR of SARS-CoV-2 variants. (A) Schematic representation of the secondary structure of the SARS-CoV-2 5'-UTR. **(B)** Top: SHAPE reactivity comparison of probed mutation C241U (orange line) and the wt (black line). Area of interest is highlighted in gray. Bottom: Comparison of SHAPE reactivities of the area of interest of tested SARS-CoV-2 variant 5'-UTRs with (orange dots) or without (black dots) mutation C241U. Secondary structure of the apical loop of SL5b and annotated mutation. **(C)** Top: SHAPE reactivity comparison of probed variant mutations G210U/C241U (orange line) and the wt (black line). Area of interest is highlighted in gray. Bottom: Comparison of SHAPE reactivities of the area of interest of tested SARS-CoV-2 variant 5'-UTRs with (orange dots) or without (black dots) mutation G210U/C241U. **(D)** Top: Scheme of the dual luciferase reporter construct for transfection in HEK293 cells. Bottom: Dual luciferase reporter assay with full-length SARS-CoV-2 5'-UTR wt and variants (n=5). Firefly luciferase activity was normalized to Renilla luciferase activity (ratio fLuc/hRluc), which served as an internal control. Statistical significance was calculated using Student's t-test (two-tailed, paired), (*) p-value <0.05, ns = not significant.

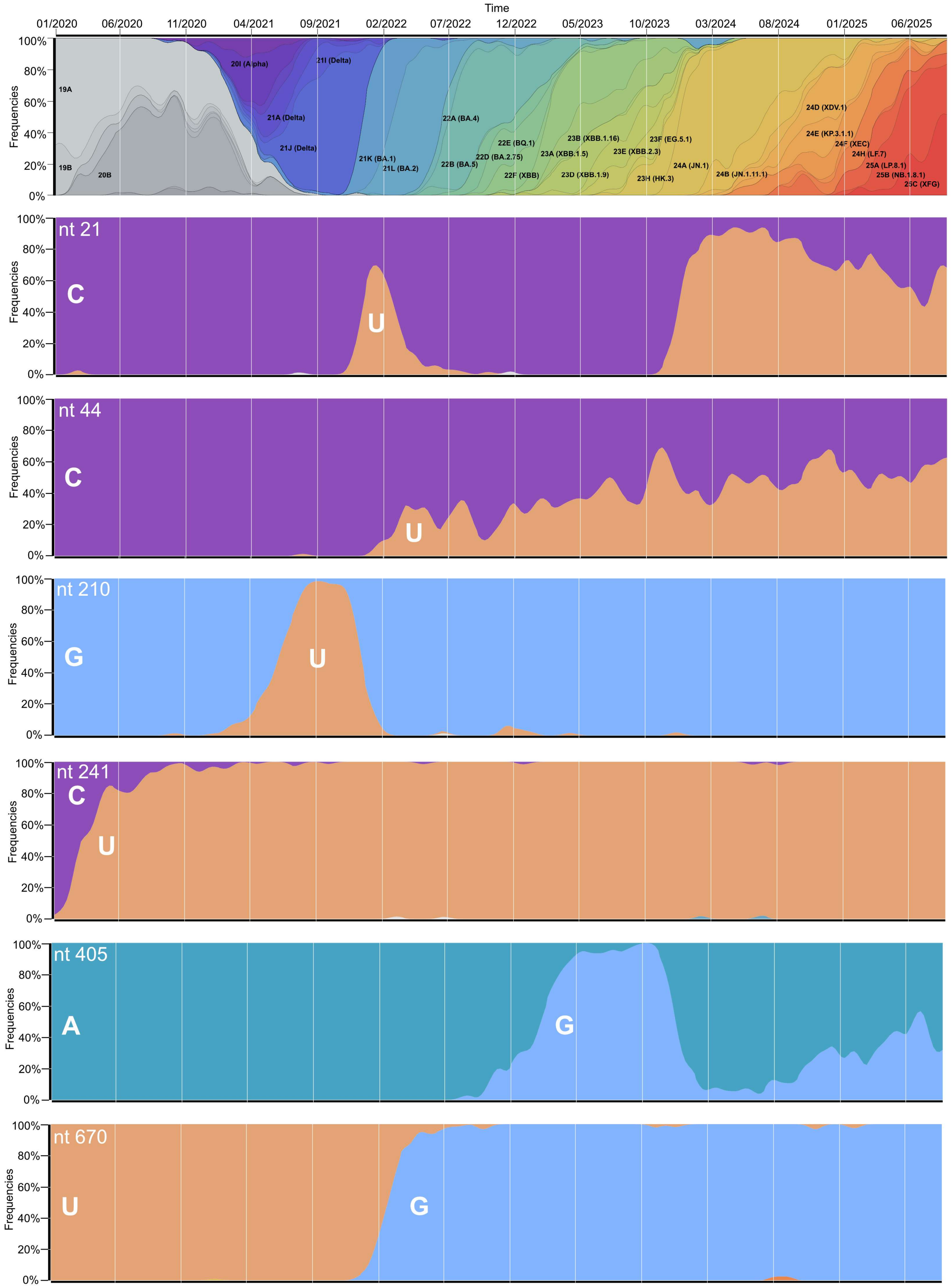

Figure S3. Frequencies of SARS-CoV-2 variants and selected mutations. Occurrence of mutations in the 5'-UTR at positions 21, 44, 210, 241 as well as in the coding region of nsp1, positions 405 and 670. Screenshots were taken from (1) and modified.

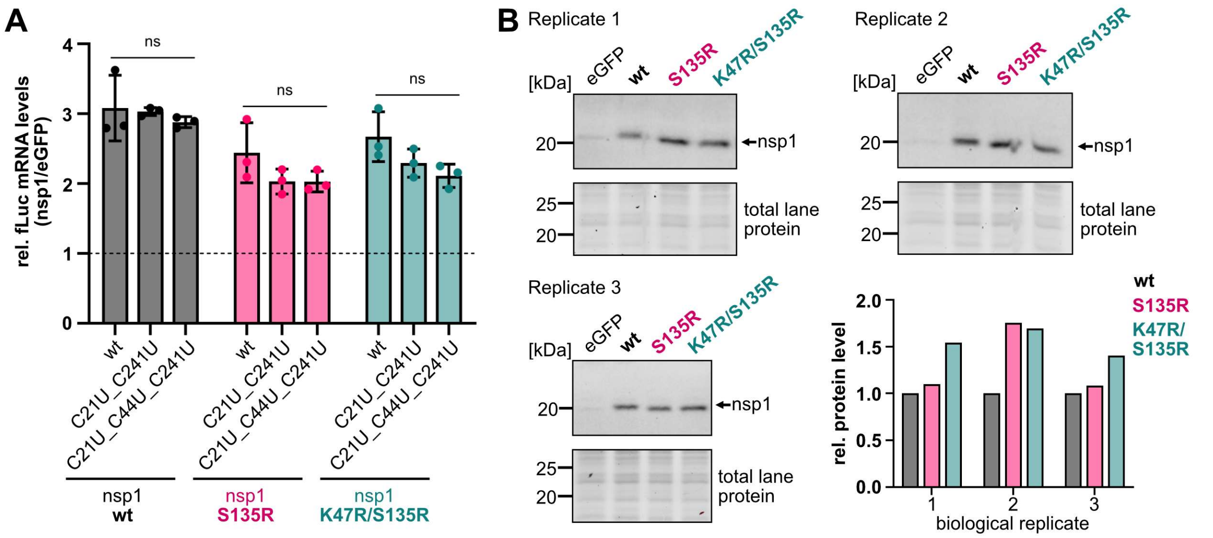

Figure S4. Relative mRNA levels of full-length SARS-CoV-2 5'-UTR reporters and relative nsp1 protein levels. (A) Relative firefly mRNA levels of dual luciferase reporters with depicted SARS-CoV-2 5'-UTR variants in HEK293 cells in co-expression with nsp1 wt or mutants (n=3). Statistical significance was calculated using Student's t-test (two-tailed, paired), ns = not significant. **(B)** Western blots of HEK293 cell lysates expressing eGFP or SARS-CoV-2 nsp1 wt, mutant S135R or mutant K47R/S135R. Each blot represents one biological replicate (n=3). Quantification of western blots was performed for each replicate in reference to the total lane protein and relative protein level was calculated by normalizing the nsp1 mutant S135R and mutant K47R/S135R to the wt for each replicate (n=3).

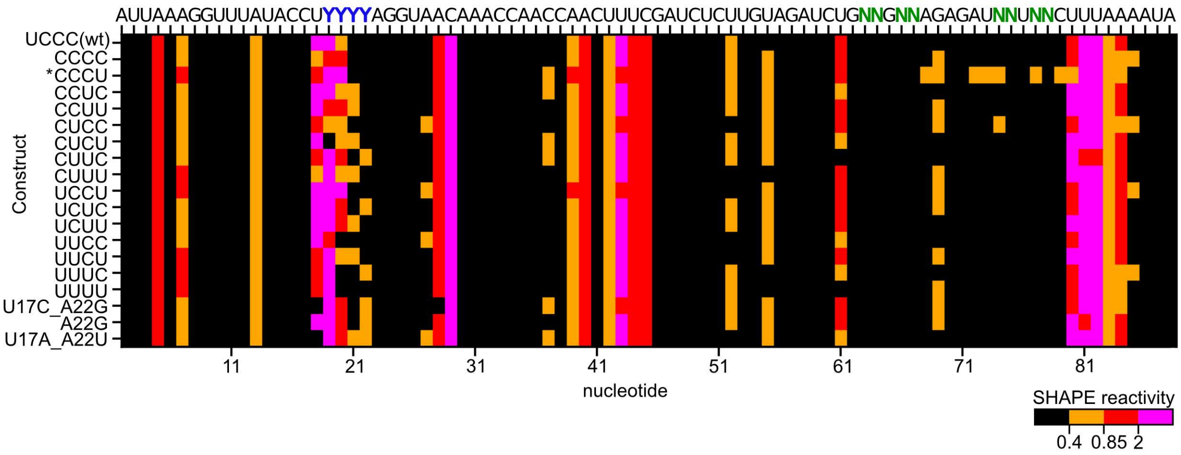

Figure S5. Full-length heatmap of SHAPE-MaP data of SL1_YYYY constructs and closing base pair mutants. Y = pyrimidines. N = barcode nucleotide according to Table S2. *The increased reactivity observed for nucleotides 72-74 and 77 is in accordance with an alternative conformation of the barcode stem, which does not affect formation of SL1 or SL2.

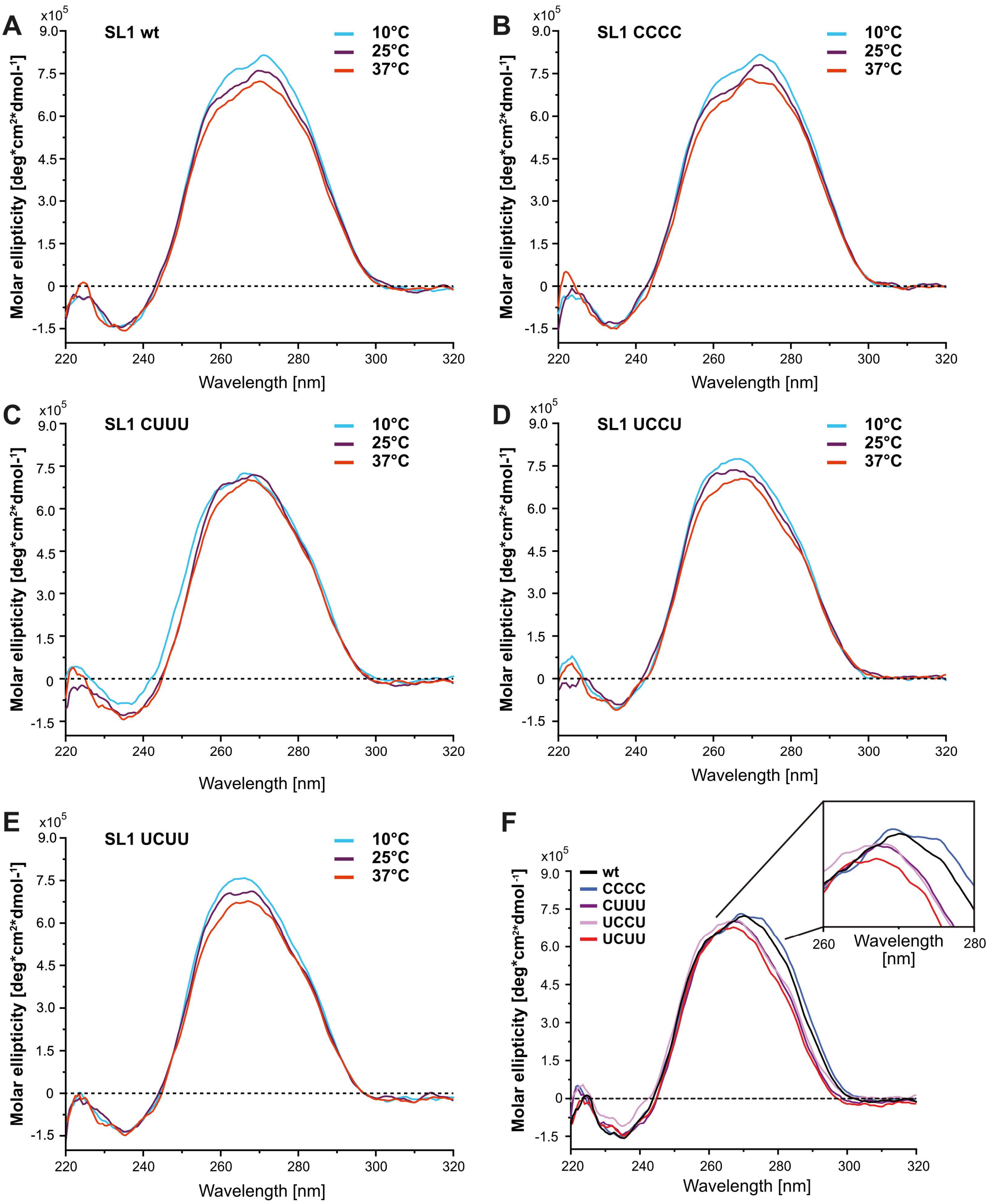

Figure S6. CD spectra of SL1 wt and the depicted SL1 mutants at different temperatures. Spectra were recorded in a wavelength range of 320-220 nm at three different temperatures (10°C, 25°C, 37°C). **(A)-(E)** CD spectra of the different RNAs, SL1 wt, CCCC, CUUU, UCCU, UCUU, recorded at 10°C (blue), 25°C (violet), and 37°C (red). **(F)** Overlay of the CD spectra of all the analyzed variants, recorded at 37°C, for direct comparison, highlighting the mutation-dependent shifts in the CD_{max} signal.

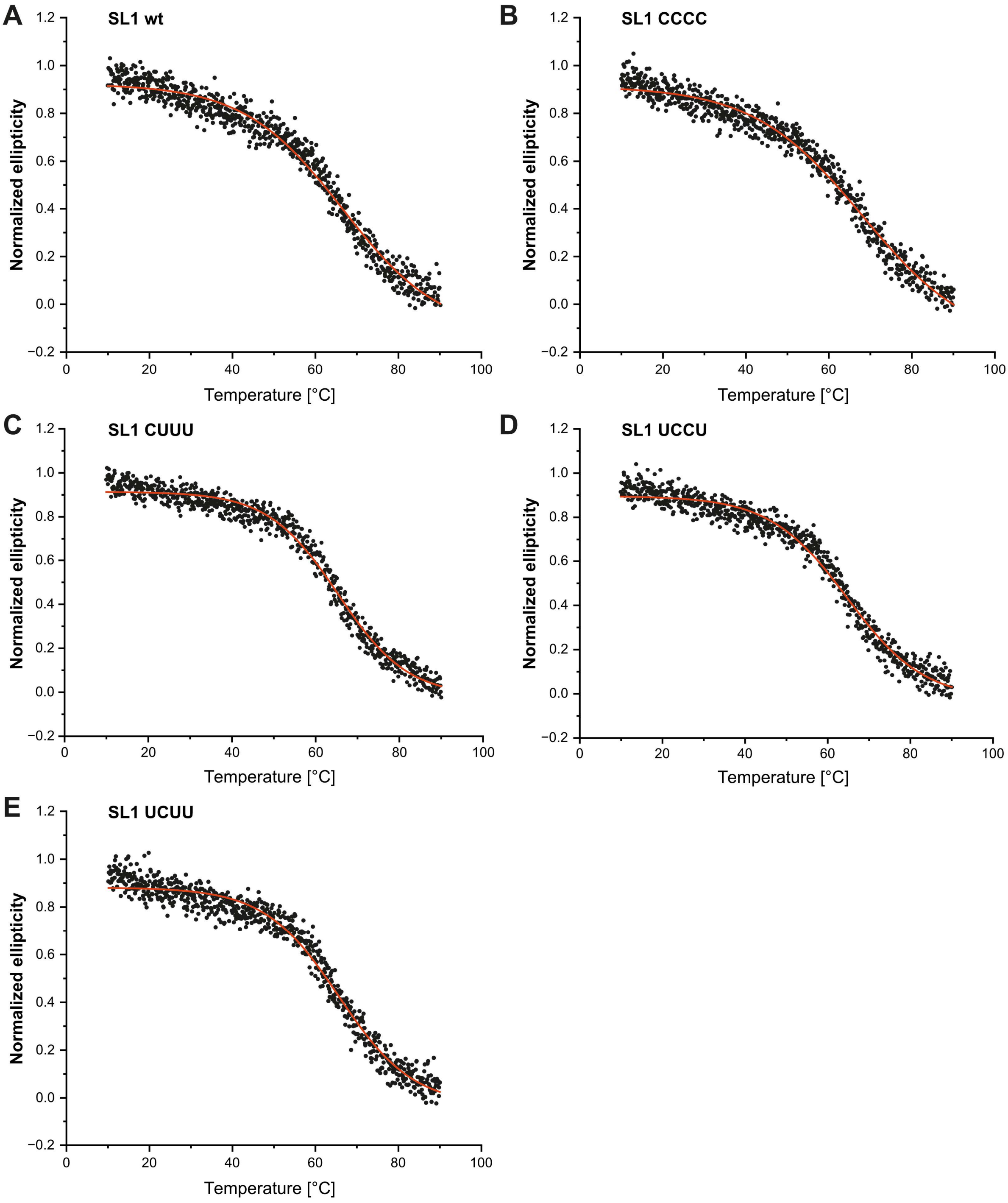

Figure S7. CD melting curves were recorded at the wavelength corresponding to the highest detected CD signal at 25°C. Measurements were performed from 10°C to 90°C, with a sampling rate of 0.5°C/min. Melting points were determined via nonlinear regression with a sigmoidal fit function (equation [1]). **(A)-(E)** CD melting curves of the different RNAs: SL1 wt, CCCC, CUUU, UCCU, UCUU.

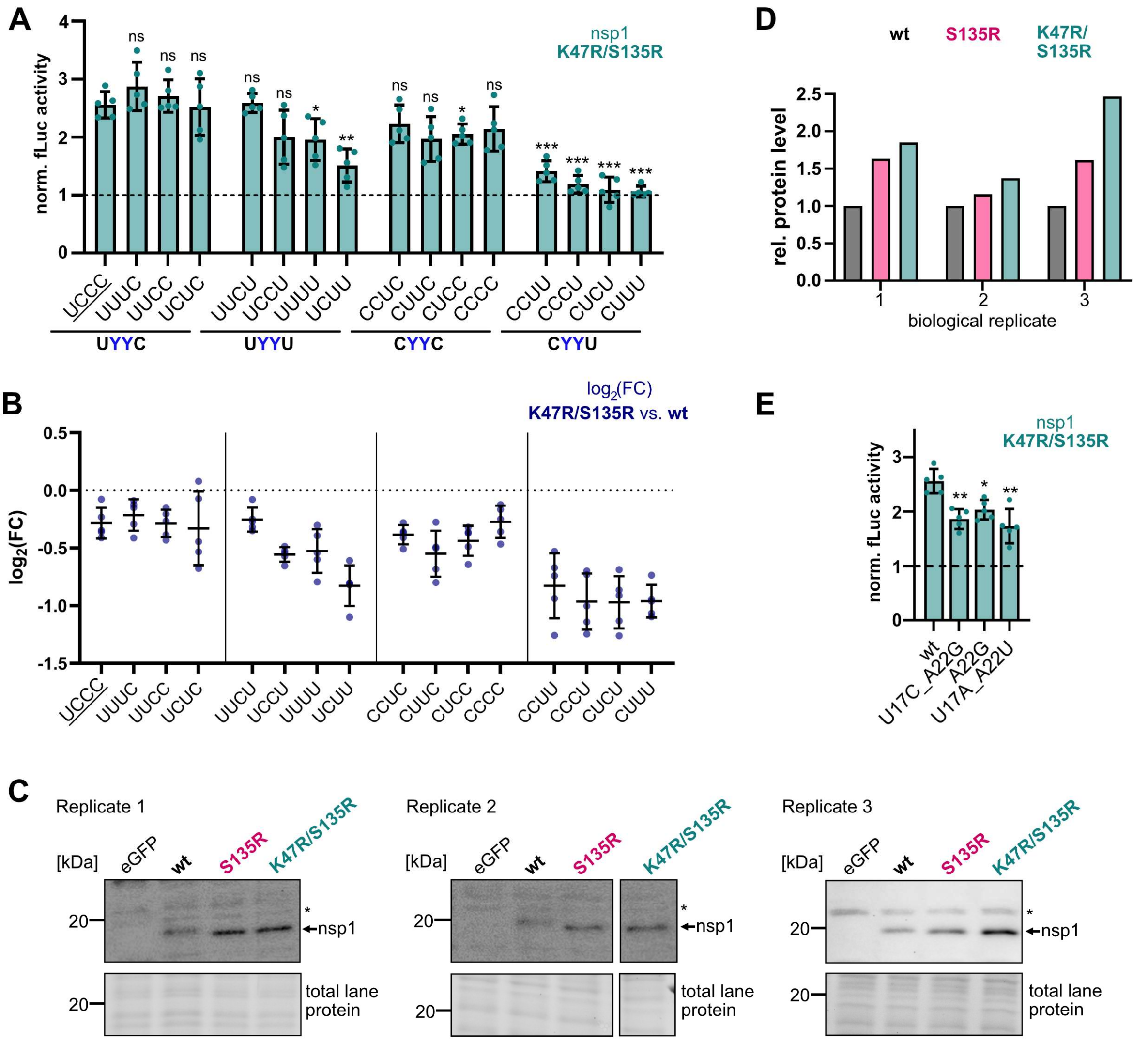

Figure S8. Activity of SARS-CoV-2 nsp1 double mutant K47R/S135R in response to the pyrimidine composition of the SARS-CoV-2 SL1 apical loop. (A) Single luciferase assay in HEK293 cells with SL1_YYYY constructs and SARS-CoV-2 nsp1 K47R/S135R (n=5). Firefly luciferase activity with SARS-CoV-2 nsp1 mutant K47R/S135R was normalized to the firefly activity of the respective construct with eGFP co-transfection. The dashed line represents the firefly luciferase activity with eGFP. Statistical significance was calculated using Student's t-test (two-tailed, paired), (***) p-value <0.001, (**) p-value <0.01, (*) p-value <0.05, ns = not significant. **(B)** Log_2(FC) of normalized luciferase activity of nsp1 mutant S135R to nsp1 wt. **(C)** Western blots of HEK293 cell lysates expressing eGFP or SARS-CoV-2 nsp1 wt, mutant S135R or mutant K47R/S135R. Top, middle and bottom blot each correspond to one biological replicate (n=3). (*) = unspecific protein. **(D)** Quantification of western blots shown in (C). Relative protein level was calculated by normalizing the nsp1 mutant S135R and mutant K47R/S135R to the wt (n=3). **(E)** Single luciferase assay in HEK293 cells with SARS-CoV-2 SL1 closing base pair constructs and SARS-CoV-2 nsp1 mutant K47R/S135R (n=5). Normalized firefly activity and statistical significance was calculated as described in (A).

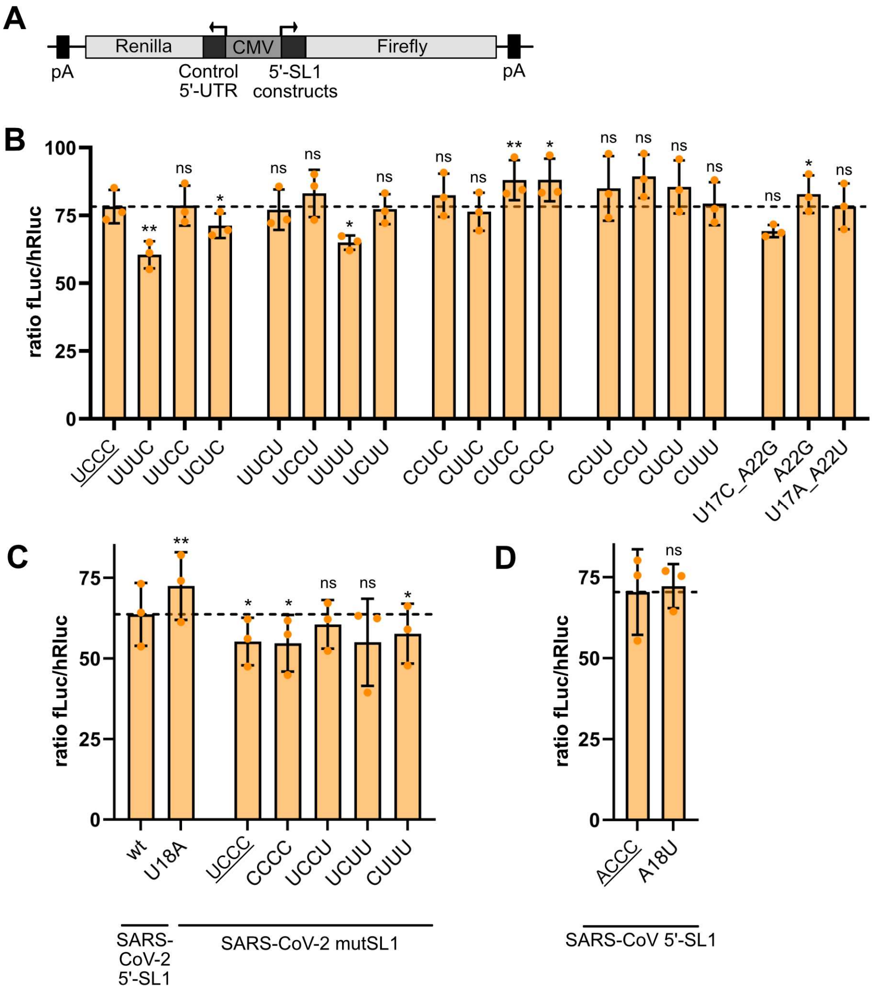

Figure S9. Relative translation efficiency in dual luciferase reporter assays of SL1 constructs. (A) Schematic representation of the dual luciferase reporter construct for transfection in HEK293 cells. **(B)** Dual luciferase reporter assay in HEK293 cells with SARS-CoV-2 SL1_YYYY constructs and closing base pair mutants (n=3). The wt apical loop sequence is underlined. Firefly luciferase activity was normalized to *Renilla* luciferase activity. Statistical significance was calculated using Student's t-test (two-tailed, paired), (**) p-value <0.01, (*) p-value <0.05, ns = not significant. **(C)** Dual luciferase reporter assay in HEK293 cells with mutated SARS-CoV-2 SL1 constructs (mutSL1) and loop compositions (n=3). Firefly luciferase activity and statistical significance was calculated as in (B). The wt apical loop sequence is underlined. **(D)** Dual luciferase reporter assay in HEK293 cells with SARS-CoV SL1 wt and mutant A18U (n=3). The wt apical loop sequence is underlined. Firefly luciferase activity and statistical significance was calculated as in (B).

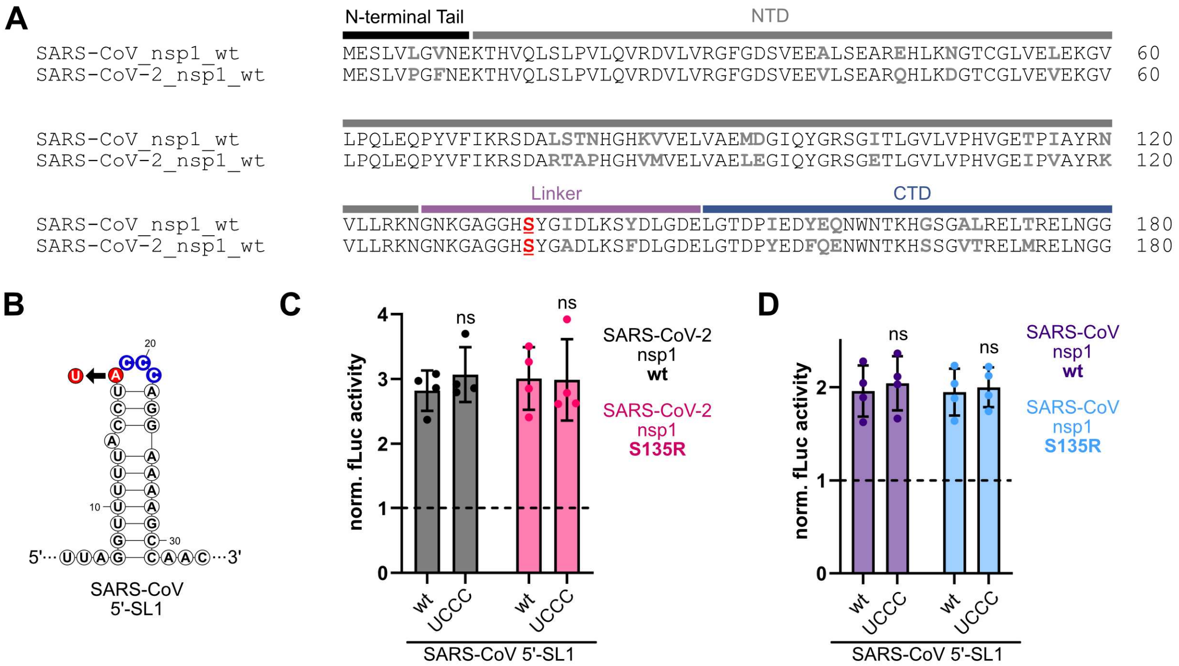

Figure S10. Activity of SARS-CoV-2 and SARS-CoV nsp1 wt and mutants with SARS-CoV SL1. **(A)** Alignment of SARS-CoV nsp1 wt and SARS-CoV-2 nsp1 wt using Clustal Omega (2). Amino acids differing between SARS-CoV and SARS-CoV-2 nsp1 are highlighted in gray. Annotation of protein domains as described previously (15). **(B)** Secondary structure of SARS-CoV SL1. **(C)** Single luciferase assay in HEK293 cells with SARS-CoV SL1 wt and mutant A18U (UCCC) and SARS-CoV-2 nsp1 wt and mutant S135R (n=4). Firefly luciferase activity was calculated by normalizing firefly activity with SARS-CoV-2 nsp1 wt or mutant S135R to the firefly activity of the respective construct with eGFP co-transfection. The dashed line represents the firefly luciferase activity with eGFP. Statistical significance was calculated using Student's t-test (two-tailed, paired), ns = not significant. **(D)** Single luciferase assay in HEK293 cells with SARS-CoV SL1 wt and mutant A18U and SARS-CoV-2 nsp1 wt and mutant S135R (n=4). Normalized firefly luciferase activity and statistical significance was calculated as described in (C).

Supplementary Tables

Table S1. Full-length RNA sequences of coronaviral 5'-UTRs for structural probing using SHAPE-MaP.

**5'-UTR**	**Full-length RNA sequence (5'->3')**
SARS-CoV-2 wt<br>(NC_045512)	AUUAAAGGUUUAUACCUUCCCAGGUAACAAACCAACCAACUUUCG<br>AUCUCUUGUAGAUCUGUUCUCUAAACGAACUUUAAAAUCUGUGUG<br>GCUGUCACUCGGCUGCAUGCUUAGUGCACUCACGCAGUAUAAUUA<br>AUAACUAAUUACUGUCGUUGACAGGACACGAGUAACUCGUCUAUC<br>UUCUGCAGGCUGCUUACGGUUUCGUCCGUGUUGCAGCCGAUCAUC<br>AGCACAUCUAGGUUUCGUCCGGGUGUGACCGAAAGGUAAGAUGGA<br>GAGCCUUGUCCCUGGUUUCAACGAGAA
SARS-CoV-2<br>C21U_C241U	AUUAAAGGUUUAUACCUUCC**U**AGGUAACAAACCAACCAACUUUCG<br>AUCUCUUGUAGAUCUGUUCUCUAAACGAACUUUAAAAUCUGUGUG<br>GCUGUCACUCGGCUGCAUGCUUAGUGCACUCACGCAGUAUAAUUA<br>AUAACUAAUUACUGUCGUUGACAGGACACGAGUAACUCGUCUAUC<br>UUCUGCAGGCUGCUUACGGUUUCGUCCGUGUUGCAGCCGAUCAUC<br>AGCACAUCUAGGUUU**U**GUCCGGGUGUGACCGAAAGGUAAGAUGG<br>AGAGCCUUGUCCCUGGUUUCAACGAGAA
SARS-CoV-2<br>C44U_C241U	AUUAAAGGUUUAUACCUUCCCAGGUAACAAACCAACCAACUUU**U**G<br>AUCUCUUGUAGAUCUGUUCUCUAAACGAACUUUAAAAUCUGUGUG<br>GCUGUCACUCGGCUGCAUGCUUAGUGCACUCACGCAGUAUAAUUA<br>AUAACUAAUUACUGUCGUUGACAGGACACGAGUAACUCGUCUAUC<br>UUCUGCAGGCUGCUUACGGUUUCGUCCGUGUUGCAGCCGAUCAUC<br>AGCACAUCUAGGUUU**U**GUCCGGGUGUGACCGAAAGGUAAGAUGG<br>AGAGCCUUGUCCCUGGUUUCAACGAGAA
SARS-CoV-2<br>C21U_C44U_C241U	AUUAAAGGUUUAUACCUUCC**U**AGGUAACAAACCAACCAACUUU**U**G<br>AUCUCUUGUAGAUCUGUUCUCUAAACGAACUUUAAAAUCUGUGUG<br>GCUGUCACUCGGCUGCAUGCUUAGUGCACUCACGCAGUAUAAUUA<br>AUAACUAAUUACUGUCGUUGACAGGACACGAGUAACUCGUCUAUC<br>UUCUGCAGGCUGCUUACGGUUUCGUCCGUGUUGCAGCCGAUCAUC<br>AGCACAUCUAGGUUU**U**GUCCGGGUGUGACCGAAAGGUAAGAUGG<br>AGAGCCUUGUCCCUGGUUUCAACGAGAA
SARS-CoV-2<br>G210U_C241U	AUUAAAGGUUUAUACCUUCCCAGGUAACAAACCAACCAACUUUCG<br>AUCUCUUGUAGAUCUGUUCUCUAAACGAACUUUAAAAUCUGUGUG<br>GCUGUCACUCGGCUGCAUGCUUAGUGCACUCACGCAGUAUAAUUA<br>AUAACUAAUUACUGUCGUUGACAGGACACGAGUAACUCGUCUAUC<br>UUCUGCAGGCUGCUUACGGUUUCGUCCGU**U**UUGCAGCCGAUCAUC<br>AGCACAUCUAGGUUU**U**GUCCGGGUGUGACCGAAAGGUAAGAUGG<br>AGAGCCUUGUCCCUGGUUUCAACGAGAA

SARS-CoV-2 C241U	AUUAAAGGUUUAUACCUUCCCAGGUAACAAACCAACCAACUUUCG AUCUCUUGUAGAUCUGUUCUCUAAACGAACUUUAAAAUCUGUGUG GCUGUCACUCGGCUGCAUGCUUAGUGCACUCACGCAGUAUAAUUA AUAACUAAUUACUGUCGUUGACAGGACACGAGUAACUCGUCUAUC UUCUGCAGGCUGCUUACGGUUUCGUCCGUGUUGCAGCCGAUCAUC AGCACAUCUAGGUUU**U**GUCCGGGUGUGACCGAAAGGUAAGAUGG AGAGCCUUGUCCCUGGUUUCAACGAGAA

Table S2. Overview of barcode sequences and RNA sequences of each SL1_YYYY variant for structural probing using SHAPE-MaP.

Tetraloop Sequence	Unique barcode 1	Unique barcode 2	Full-length RNA sequence (5'->3')
CCCC	ACAU	AUGU	AUUAAAGGUUUAUACCUCCCCAGGUAACAAAC CAACCAACUUUCGAUCUCUUGUAGAUCUGACG AUAGAGAUAUUGUCUUUAAAAUA
CCCU	AACU	AGUU	AUUAAAGGUUUAUACCUCCCUAGGUAACAAAC CAACCAACUUUCGAUCUCUUGUAGAUCUGAAG CUAGAGAUAGUUUCUUUAAAAUA
CCUC	ACGC	GCGU	AUUAAAGGUUUAUACCUCCUCAGGUAACAAAC CAACCAACUUUCGAUCUCUUGUAGAUCUGACG GCAGAGAUGCUGUCUUUAAAAUA
CCUU	AGCC	GGCU	AUUAAAGGUUUAUACCUCCUUAGGUAACAAAC CAACCAACUUUCGAUCUCUUGUAGAUCUGAGG CCAGAGAUGGUCUCUUUAAAAUA
CUCC	AUGU	ACAU	AUUAAAGGUUUAUACCUCUCCAGGUAACAAAC CAACCAACUUUCGAUCUCUUGUAGAUCUGAUG GUAGAGAUACUAUCUUUAAAAUA
CUCU	CAGC	GCUG	AUUAAAGGUUUAUACCUCUCUAGGUAACAAAC CAACCAACUUUCGAUCUCUUGUAGAUCUGCAG GCAGAGAUGCUGUCUUUAAAAUA
CUUC	CCGG	CCGG	AUUAAAGGUUUAUACCUCUUCAGGUAACAAAC CAACCAACUUUCGAUCUCUUGUAGAUCUGCCG GGAGAGAUCCUGGCUUUAAAAUA
CUUU	CGUU	AACG	AUUAAAGGUUUAUACCUCUUUAGGUAACAAAC CAACCAACUUUCGAUCUCUUGUAGAUCUGCGG UUAGAGAUAAUCGCUUUAAAAUA
UCCC	CUAC	GUAG	AUUAAAGGUUUAUACCUUCCCAGGUAACAAAC CAACCAACUUUCGAUCUCUUGUAGAUCUGCUG ACAGAGAUGUUAGCUUUAAAAUA

UCCU	GAAU	AUUC	AUUAAAGGUUUAUACCUUCCUAGGUAACAAAC CAACCAACUUUCGAUCUCUUGUAGAUCUGGAG AUAGAGAUAUUUCCUUUAAAAUA
UCUC	GCAA	UUGC	AUUAAAGGUUUAUACCUUCUCAGGUAACAAAC CAACCAACUUUCGAUCUCUUGUAGAUCUGGCG AAAGAGAUUUUGCCUUUAAAAUA
UCUU	GCUG	CAGC	AUUAAAGGUUUAUACCUUCUUAGGUAACAAAC CAACCAACUUUCGAUCUCUUGUAGAUCUGGCG UGAGAGAUCAUGCCUUUAAAAUA
UUCC	GUAG	CUAC	AUUAAAGGUUUAUACCUUUCCAGGUAACAAAC CAACCAACUUUCGAUCUCUUGUAGAUCUGGUG AGAGAGAUCUUACCUUUAAAAUA
UUCU	GUUC	GAAC	AUUAAAGGUUUAUACCUUUCUAGGUAACAAAC CAACCAACUUUCGAUCUCUUGUAGAUCUGGUG UCAGAGAUGAUACCUUUAAAAUA
UUUC	UAAC	GUUA	AUUAAAGGUUUAUACCUUUUCAGGUAACAAAC CAACCAACUUUCGAUCUCUUGUAGAUCUGUAG ACAGAGAUGUUUACUUUAAAAUA
UUUU	UAUC	GAUA	AUUAAAGGUUUAUACCUUUUUAGGUAACAAAC CAACCAACUUUCGAUCUCUUGUAGAUCUGUAG UCAGAGAUGAUUACUUUAAAAUA
**Closing base-pair mutations**			
U17C, A22G	UCUU	AAGA	AUUAAAGGUUUAUACCCUCCCGGGUAACAAAC CAACCAACUUUCGAUCUCUUGUAGAUCUGUCG UUAGAGAUAAUGACUUUAAAAUA
A22G	UGCU	AGCA	AUUAAAGGUUUAUACCUUCCCGGGUAACAAAC CAACCAACUUUCGAUCUCUUGUAGAUCUGUGG CUAGAGAUAGUCACUUUAAAAUA
U17A, A22U	UGGC	GCCA	AUUAAAGGUUUAUACCAUCCCUGGUAACAAAC CAACCAACUUUCGAUCUCUUGUAGAUCUGUGG GCAGAGAUGCUCACUUUAAAAUA

Table S3. DNA sequences of full-length SARS-CoV-2 5'-UTRs in the dual luciferase reporter gene (hRluc/fLuc) plasmids with bidirectional CMV promoter. First nucleotide is the transcription start site downstream of CMV promoter. The start codon of the open reading frame is indicated in bold.

**5'-UTR**	**Sequence (5'->3')**
Control 5'-UTR upstream of hRluc	GTCAGATCGCCTGGAGAATTCACCGGTCATAAGCCGCGGGGGCCCAGATCT**ATG**
SARS-CoV-2 5'-UTR wt upstream of fLuc	ATTAAAGGTTTATACCTTCCCAGGTAACAAACCAACCAACTTTCGATCTCTTGTAGATCTGTTCTCTAAACGAACTTTAAAATCTGTGTGGCTGTCACTCGGCTGCATGCTTAGTGCACTCACGCAGTATAATTAATAACTAATTACTGTCGTTGACAGGACACGAGTAACTCGTCTATCTTCTGCAGGCTGCTTACGGTTTCGTCCGTGTTGCAGCCGATCATCAGCACATCTAGGTTTCGTCCGGGTGTGACCGAAAGGTAAG**ATG**GAGAGCCTTGTCCCTGGTTTCAACGAGAAAGAA
SARS-CoV-2 5'-UTR C21U_C241U upstream of fLuc	ATTAAAGGTTTATACCTTCCTAGGTAACAAACCAACCAACTTTCGATCTCTTGTAGATCTGTTCTCTAAACGAACTTTAAAATCTGTGTGGCTGTCACTCGGCTGCATGCTTAGTGCACTCACGCAGTATAATTAATAACTAATTACTGTCGTTGACAGGACACGAGTAACTCGTCTATCTTCTGCAGGCTGCTTACGGTTTCGTCCGTGTTGCAGCCGATCATCAGCACATCTAGGTTTTGTCCGGGTGTGACCGAAAGGTAAG**ATG**GAGAGCCTTGTCCCTGGTTTCAACGAGAAAGAA
SARS-CoV-2 5'-UTR C44U_C241U upstream of fLuc	ATTAAAGGTTTATACCTTCCCAGGTAACAAACCAACCAACTTTTGATCTCTTGTAGATCTGTTCTCTAAACGAACTTTAAAATCTGTGTGGCTGTCACTCGGCTGCATGCTTAGTGCACTCACGCAGTATAATTAATAACTAATTACTGTCGTTGACAGGACACGAGTAACTCGTCTATCTTCTGCAGGCTGCTTACGGTTTCGTCCGTGTTGCAGCCGATCATCAGCACATCTAGGTTTTGTCCGGGTGTGACCGAAAGGTAAG**ATG**GAGAGCCTTGTCCCTGGTTTCAACGAGAAAGAA
SARS-CoV-2 5'-UTR C21U_C44U_C241U upstream of fLuc	ATTAAAGGTTTATACCTTCCTAGGTAACAAACCAACCAACTTTTGATCTCTTGTAGATCTGTTCTCTAAACGAACTTTAAAATCTGTGTGGCTGTCACTCGGCTGCATGCTTAGTGCACTCACGCAGTATAATTAATAACTAATTACTGTCGTTGACAGGACACGAGTAACTCGTCTATCTTCTGCAGGCTGCTTACGGTTTCGTCCGTGTTGCAGCCGATCATCAGCACATCTAGGTTTTGTCCGGGTGTGACCGAAAGGTAAG**ATG**GAGAGCCTTGTCCCTGGTTTCAACGAGAAAGAA
SARS-CoV-2 5'-UTR G210U_C241U upstream of fLuc	ATTAAAGGTTTATACCTTCCCAGGTAACAAACCAACCAACTTTCGATCTCTTGTAGATCTGTTCTCTAAACGAACTTTAAAATCTGTGTGGCTGTCACTCGGCTGCATGCTTAGTGCACTCACGCAGTATAATTAATAACTAATTACTGTCGTTGACAGGACACGAGTAACTCGTCTATCTTCTGCA

	GGCTGCTTACGGTTTCGTCCGTTTTGCAGCCGATCATCAGCACATCT AGGTTTTGTCCGGGTGTGACCGAAAGGTAAG**ATG**GAGAGCCTTGT CCCTGGTTTCAACGAGAAAGAA
SARS-CoV-2 5'-UTR C241U upstream of fLuc	ATTAAAGGTTTATACCTTCCCAGGTAACAAACCAACCAACTTTCGAT CTCTTGTAGATCTGTTCTCTAAACGAACTTTAAAATCTGTGTGGCTG TCACTCGGCTGCATGCTTAGTGCACTCACGCAGTATAATTAATAACT AATTACTGTCGTTGACAGGACACGAGTAACTCGTCTATCTTCTGCA GGCTGCTTACGGTTTCGTCCGTGTTGCAGCCGATCATCAGCACATC TAGGTTTTGTCCGGGTGTGACCGAAAGGTAAG**ATG**GAGAGCCTTG TCCCTGGTTTCAACGAGAAAGAA

Table S4. DNA sequences of SL1 constructs in the single luciferase reporter gene (fLuc) and dual luciferase reporter gene (hRluc/fLuc) plasmids. First nucleotide is the transcription start site downstream of CMV promoter. The start codon of the open reading frame is indicated in bold.

**5'-UTR**	**Sequence (5'->3')**
Control 5'-UTR upstream of hRluc	GTCAGATCGCCTGGAGAATTCACCGGTCATAAGCCGCGGGGGCC CAGATCT**ATG**
5'-SL1 CCCC upstream of fLuc	ATTAAAGGTTTATACCTCCCCAGGTAACAAACCAACCAACTTTCA CC**ATG**
5'-SL1 CCCU upstream of fLuc	ATTAAAGGTTTATACCTCCCTAGGTAACAAACCAACCAACTTTCA CC**ATG**
5'-SL1 CCUC upstream of fLuc	ATTAAAGGTTTATACCTCCTCAGGTAACAAACCAACCAACTTTCA CC**ATG**
5'-SL1 CCUU upstream of fLuc	ATTAAAGGTTTATACCTCCTTAGGTAACAAACCAACCAACTTTCAC C**ATG**
5'-SL1 CUCC upstream of fLuc	ATTAAAGGTTTATACCTCTCCAGGTAACAAACCAACCAACTTTCA CC**ATG**
5'-SL1 CUCU upstream of fLuc	ATTAAAGGTTTATACCTCTCTAGGTAACAAACCAACCAACTTTCAC C**ATG**
5'-SL1 CUUC upstream of fLuc	ATTAAAGGTTTATACCTCTTCAGGTAACAAACCAACCAACTTTCAC C**ATG**
5'-SL1 CUUU upstream of fLuc	ATTAAAGGTTTATACCTCTTTAGGTAACAAACCAACCAACTTTCAC C**ATG**
5'-SL1 UCCC (wt) upstream of fLuc	ATTAAAGGTTTATACCTTCCCAGGTAACAAACCAACCAACTTTCA CC**ATG**
5'-SL1 UCCU (C21U) upstream of fLuc	ATTAAAGGTTTATACCTTCCTAGGTAACAAACCAACCAACTTTCAC C**ATG**

5'-SL1 UCUC upstream of fLuc	ATTAAAGGTTTATACCTTCTCAGGTAACAAACCAACCAACTTTCACC**ATG**
5'-SL1 UCUU upstream of fLuc	ATTAAAGGTTTATACCTTCTTAGGTAACAAACCAACCAACTTTCACC**ATG**
5'-SL1 UUCC upstream of fLuc	ATTAAAGGTTTATACCTTTCCAGGTAACAAACCAACCAACTTTCACC**ATG**
5'-SL1 UUCU upstream of fLuc	ATTAAAGGTTTATACCTTTCUAGGTAACAAACCAACCAACTTTCACC**ATG**
5'-SL1 UUUC upstream of fLuc	ATTAAAGGTTTATACCTTTTCAGGTAACAAACCAACCAACTTTCACC**ATG**
5'-SL1 UUUU upstream of fLuc	ATTAAAGGTTTATACCTTTTTAGGTAACAAACCAACCAACTTTCACC**ATG**
5'-SL1 U17C, A22G upstream of fLuc	ATTAAAGGTTTATACCCTCCCGGGTAACAAACCAACCAACTTTCACC**ATG**
5'-SL1 A22G upstream of fLuc	ATTAAAGGTTTATACCTTCCCGGGTAACAAACCAACCAACTTTCACC**ATG**
5'-SL1 U17A, A22U upstream of fLuc	ATTAAAGGTTTATACCATCCCUGGTAACAAACCAACCAACTTTCACC**ATG**
5'-SL1 U18A upstream of fLuc	ATTAAAGGTTTATACCTACCCAGGTAACAAACCAACCAACTTTCACC**ATG**
5'-mutSL1 UCCC (wt) upstream of fLuc	ATTAAAGGTTTATACTTTCCCAGATAACAAACCAACCAACTTTCACC**ATG**
5'-mutSL1 UCCU (C21U) upstream of fLuc	ATTAAAGGTTTATACTTTCCTAGATAACAAACCAACCAACTTTCACC**ATG**
5'-mutSL1 CCCC upstream of fLuc	ATTAAAGGTTTATACTTCCCCAGATAACAAACCAACCAACTTTCACC**ATG**
5'-mutSL1 UCUU upstream of fLuc	ATTAAAGGTTTATACTTTCTTAGATAACAAACCAACCAACTTTCACC**ATG**
5'-mutSL1 CUUU upstream of fLuc	ATTAAAGGTTTATACTTCTTTAGATAACAAACCAACCAACTTTCACC**ATG**
SARS-CoV 5'-SL1 wt upstream of fLuc	ATATTAGGTTTTTACCTACCCAGGAAAAGCCAACCAACCTCACC**ATG**
SARS-CoV 5'-SL1 A18U upstream of fLuc	ATATTAGGTTTTTACCTTCCCAGGAAAAGCCAACCAACCTCACC**ATG**

Table S5. RNA sequences of SL1 wt and C21U (nt 7-33 of SARS-CoV-2 5'-SL1) constructs in NMR experiments.

Tetraloop Sequence	Full-length RNA sequence (5'->3')
UCCC	GGGUUUAUACCUUCCCAGGUAACAAACCC
UCCU	GGGUUUAUACCUUCCUAGGUAACAAACCC

Supplementary References

1. Hadfield J, Megill C, Bell SM, Huddleston J, Potter B, Callender C, Sagulenko P, Bedford T and Neher RA (2018) Nextstrain: real-time tracking of pathogen evolution. *Bioinformatics*, **34**, 4121–4123.

2. Madeira F, Madhusoodanan N, Lee J, Eusebi A, Niewielska A, Tivey ARN, Lopez R and Butcher S (2024) The EMBL-EBI Job Dispatcher sequence analysis tools framework in 2024. *Nucleic Acids Res.*, **52**, W521–W525.
